# Triplet states in the reaction center of Photosystem II†

**DOI:** 10.1039/d3sc02985a

**Published:** 2023-08-17

**Authors:** Sinjini Bhattacharjee, Frank Neese, Dimitrios A. Pantazis

**Affiliations:** a Max-Planck-Institut für Kohlenforschung Kaiser-Wilhelm-Platz 1 45470 Mülheim an der Ruhr Germany dimitrios.pantazis@kofo.mpg.de

## Abstract

In oxygenic photosynthesis sunlight is harvested and funneled as excitation energy into the reaction center (RC) of Photosystem II (PSII), the site of primary charge separation that initiates the photosynthetic electron transfer chain. The chlorophyll Chl_D1_ pigment of the RC is the primary electron donor, forming a charge-separated radical pair with the vicinal pheophytin Pheo_D1_ (Chl_D1_^+^Pheo_D1_^−^). To avert charge recombination, the electron is further transferred to plastoquinone Q_A_, whereas the hole relaxes to a central pair of chlorophylls (P_D1_P_D2_), subsequently driving water oxidation. Spin-triplet states can form within the RC when forward electron transfer is inhibited or back reactions are favored. This can lead to formation of singlet dioxygen, with potential deleterious effects. Here we investigate the nature and properties of triplet states within the PSII RC using a multiscale quantum-mechanics/molecular-mechanics (QM/MM) approach. The low-energy spectrum of excited singlet and triplet states, of both local and charge-transfer nature, is compared using range-separated time-dependent density functional theory (TD-DFT). We further compute electron paramagnetic resonance properties (zero-field splitting parameters and hyperfine coupling constants) of relaxed triplet states and compare them with available experimental data. Moreover, the electrostatic modulation of excited state energetics and redox properties of RC pigments by the semiquinone Q_A_^−^ is described. The results provide a detailed electronic-level understanding of triplet states within the PSII RC and form a refined basis for discussing primary and secondary electron transfer, charge recombination pathways, and possible photoprotection mechanisms in PSII.

## Abstract 2

Multiscale simulations in combination with time-dependent density functional theory are used to explore the energetics, localization, and spectroscopic properties of triplet states in the reaction center of Photosystem II.

## Graphical abstract

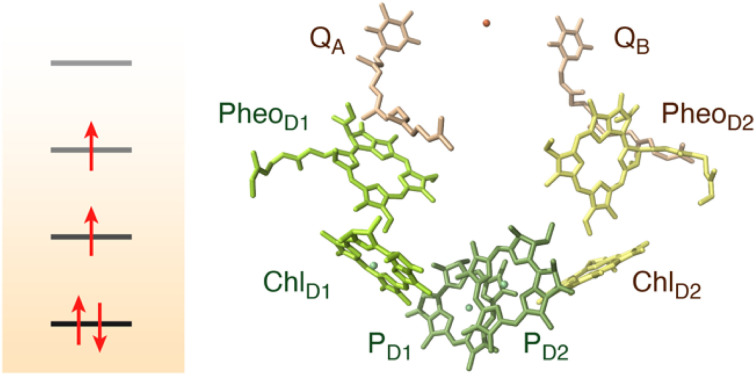

## 1. Introduction

**Fig. 1:**
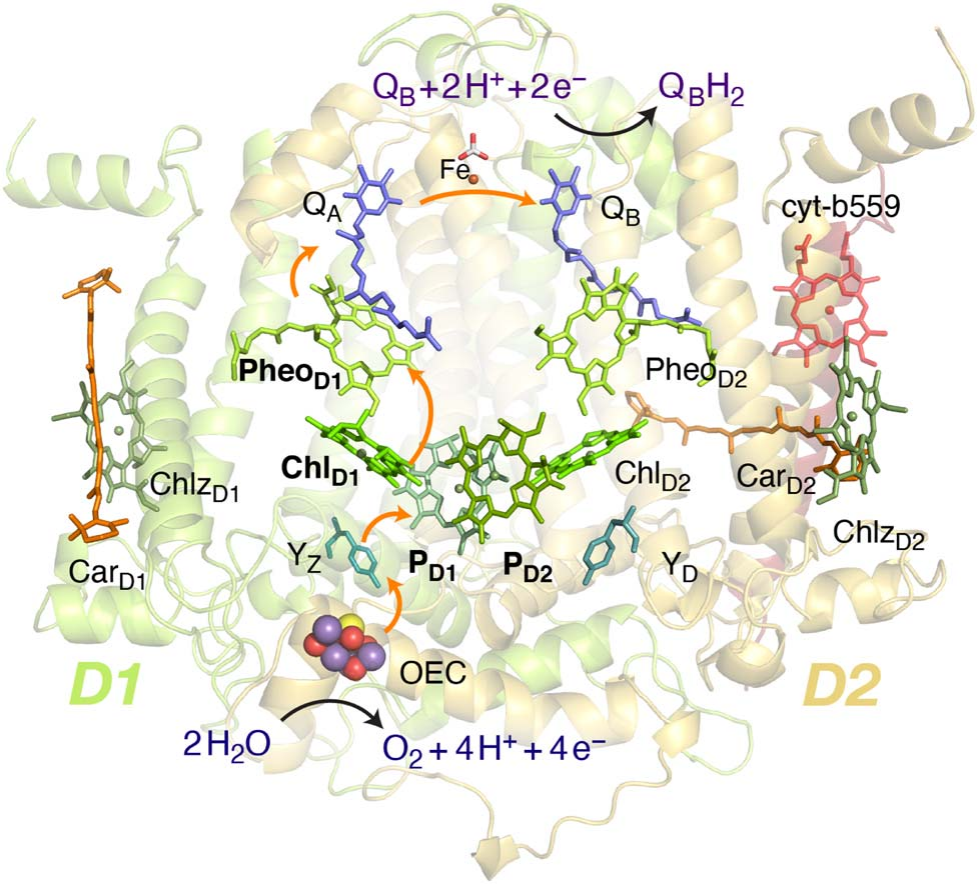
Reaction center pigments and other important cofactors, with schematic representation of electron flow along the active branch of Photosystem II.

Oxygenic photosynthesis involves a series of light-dependent electron transfer reactions which are carried out by membrane-bound pigment–protein complexes.^1^ The reactions at these energy-converting enzymes generate a transmembrane electrochemical potential gradient to drive the synthesis of ATP. The first enzyme in the photosynthetic chain is Photosystem II (PSII), a dimeric multi-subunit protein–pigment complex responsible for the four-electron oxidation of water into molecular oxygen and two-electron reduction of a mobile plastoquinone acceptor (Q_B_).^2–7^ The light-driven charge separation and the initial electron transfer events occur at the reaction center (RC) of PSII. This comprises four chlorophyll molecules, namely the P_D1_ and P_D2_ central pair flanked by the “accessory” chlorophylls Chl_D1_ and Chl_D2_, and two pheophytin molecules, Pheo_D1_ and Pheo_D2_. The RC pigments are arranged pseudo-symmetrically along the D1 and D2 heterodimeric subunits of PSII (Fig. 1) that are highly conserved across photosynthetic organisms.^8^

The excitation energy transfer from external light harvesting complexes and the internal antennae CP43 and CP47 initiates the electron transfer process along the D1 branch of the RC (Fig. 1). Charge-transfer excited states of mostly Chl_D1_^*δ*+^Pheo_D1_^*δ*−^ character are created, leading to formation of the primary charge separated radical pair Chl_D1_^+^Pheo_D1_^−^ (ref. 6 and 9–18) and the cationic charge is then distributed over the P_D1_P_D2_ pair (often referred to as P_680_^+^).^10,19–21^ This highly oxidizing radical cation (estimated *E*_m_ of 1.1–1.3 V) is the strongest known oxidant in biology and drives water oxidation at the oxygen-evolving complex.^3,22,23^ Under normal conditions, charge recombination of the initially formed radical pairs [Chl_D1_^+^Pheo_D1_^−^]^4,11,16^ (or possibly [P_D1_^+^Pheo_D1_^−^] in some reaction centers)^24^ is prevented by forward electron transfer from Pheo_D1_ to the primary plastoquinone acceptor Q_A_ within a few hundred ps. This leads to formation of the “closed RC” state with a reduced Q_A_.^5,25–29^ If the plastoquinone pool remains reduced, electron transfer from Q_A_^−^ to the mobile acceptor Q_B_ is inhibited, thus preventing further electron transfer from Pheo_D1_ to Q_A_. This can facilitate charge recombination^30–35^ within the RC and enable formation of chlorophyll triplet states prior to relaxation to the ground state.^14,30–33,36–46^ Triplet states are detrimental as they can readily generate chemically active singlet oxygen (^1^O_2_) that reacts with the protein causing oxidative stress.^47,48^ The D1 protein embeds most crucial redox cofactors in PSII, including the oxygen-evolving complex (OEC), and thus photodamage can lead to a disruption of the entire photosynthetic machinery. Correlation has been reported between ^1^O_2_ production and the extent of photodamage of the D1 protein on exposure to excess light.^49–51^ All photosynthetic organisms therefore naturally adopt intrinsic strategies of photoprotection by efficiently quenching chlorophyll triplet states either by redox active cofactors (*e.g.* Q_A_^−^ in the RC)^29,32,52^ or carotenoids^53–55^ (*e.g.* in the bacterial RC or antenna complexes), but the exact molecular mechanisms of these phenomena are not fully understood. Therefore, it is useful to have a reliable description of the nature and localization of triplet states, as an essential basis for understanding photoprotection mechanisms in PSII.

Chlorophyll triplet states, in addition to being highly reactive, serve as chemical probes to investigate primary electron transfer pathways and characterize the chemical environment of photosynthetic reaction center pigments.^44^ Electron paramagnetic resonance (EPR) and electron–nuclear double resonance (ENDOR) spectroscopies^29,37,39,44,56–69^ and other spectroscopic approaches including Fourier-transform infrared (FTIR) and optically detected magnetic resonance (ODMR)^36,38,39,51,54,70–76^ suggest that the “primary donor” triplet is located on an individual accessory chlorophyll (Chl_D1_ or Chl_D2_) at cryogenic temperatures.^30,56–58,61,77^ It has also been suggested that the triplet is partially shared with other chlorophylls at the RC at higher temperatures, but this has not been well characterized.^33,62^ It is important to note that many studies report varying observations depending on the type of preparation and conditions used, as in the case of D_1_D_2_Cyt*b*_559_ samples^60,62,78,79^ or samples with chemically reduced quinone (Q_A_^−^/Q_A_^2−^).^25,29,46,52^

Various chemical and photo-physical properties of pigments such as site energies and redox potentials^10,22,43,80,81^ are directly or indirectly controlled by the surrounding protein matrix,^82–84^ as already established in the case of charge transfer states involving the RC pigments.^12,24^ From a methodological perspective, this establishes the need for multilayer approaches to provide an accurate quantitative description of how inter-pigment and pigment–protein interactions determine spectroscopic properties. Previous excited state calculations based on time-dependent density functional theory (TD-DFT) and quantum-mechanics/molecular-mechanics (QM/MM) simulations on pigment assemblies have shown that the lowest singlet excitations in the RC are characterized by a mixture of excitonic and [Chl_D1_^*δ*+^Pheo_D1_^*δ*−^] or [P_D1_^*δ*+^Pheo_D1_^*δ*−^] charge-transfer (CT) character.^12,24,85,86^ However, a coherent description of excited and ground triplet states is lacking. The excitation profiles of all RC pigments in their triplet states are important elements for establishing possible routes of triplet delocalization^87,88^ and triplet–triplet energy transfer (T-TET) onto other pigments within the PSII core complex.^89^

In this work, we use a membrane-bound model of an entire PSII monomer as the basis for multiscale quantum-mechanics/molecular-mechanics (QM/MM) modelling to study singlet–triplet excitations as well as relaxed triplet states within the RC pigments. The quantum chemical descriptions of both local and charge-transfer excitations in oligomeric assemblies are obtained by range-separated time-dependent density functional theory (TD-DFT). We employ our QM/MM approach to also compute EPR properties of all triplet states localized on each chromophore, and compare the results with available spectroscopic data.^37,39,65^ Finally, we study how charge transfer pathways and triplet formation at the RC depend on the redox state of the primary quinone (Q_A_) acceptor and of the OEC.^61,90^ Overall, the present work contributes to a more complete understanding of the nature of triplet states within the RC of PSII, of their electronic and spectroscopic properties, and of the electrostatic control exerted by the PSII protein matrix.

## 2. Methodology

### 2.1. QM/MM setup

**Fig. 2:**
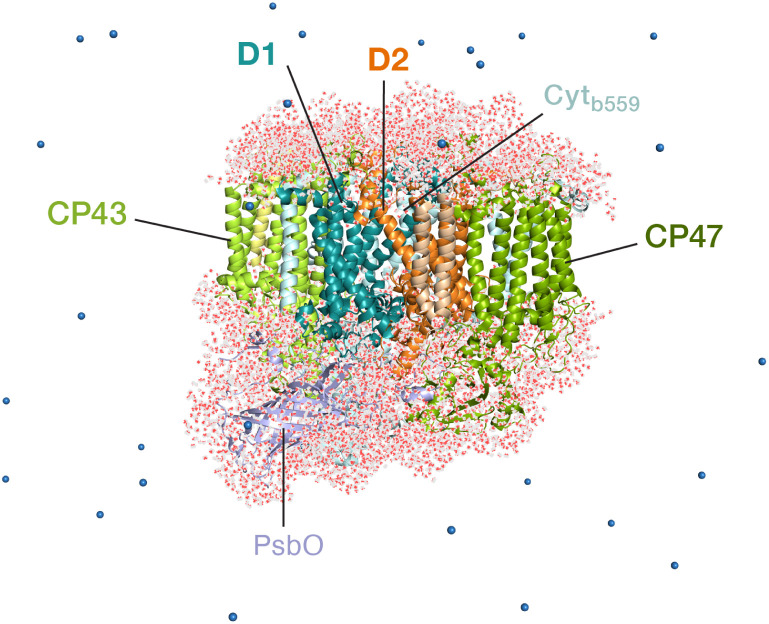
The all-atom model of the PSII monomer used for the QM/MM computations, indicating selected major protein subunits. Appropriate number of sodium ions were retained to maintain charge neutrality of the model. The overall system contains 76 056 atoms.

The classical membrane-embedded MM setup was built using the 1.9 Å resolution crystal structure of PSII from the thermophilic cyanobacterium *T. vulcanus* (PDB ID: 3WU2).^8^ In the current study we chose a snapshot that resembles the X-ray structure configuration^8,12^ from an initial MD equilibration in the work by Sirohiwal *et al.*^24^ For the QM/MM calculations we retained the complete PSII monomer and all waters around the protein (7 Å bulk-region, in total 8000 water molecules including internal cavity waters). The final atom count for this QM/MM setup was 76 056 atoms (Fig. 2). The oxidation states of the Mn ions of the OEC were assumed to correspond to the dark-stable S_1_ state of its catalytic cycle. In order to model the “closed” reaction center in the S_2_Q_A_^−^ state, the AMBER parameter file was modified with the electrostatic charges for both cofactors (Q_A_^−^ and OEC) based on the standard MK-RESP (Merz–Kollman Restrained Electrostatic Potential) methodology.^91–93^ For the semiquinone, geometry optimization was first performed at the B3LYP/def2-SVP level^94,95^ and then single-point calculations were performed at the HF/6-31G* level of theory in ORCA.^96^ In order to compute the charges on the OEC (Mn_4_CaO_5_) a small cluster model was taken including the amino acid side chains directly coordinated to each metal site. The OEC was then modelled in the S_2_ state of the Kok–Joliot cycle, *i.e.* with formal oxidation states Mn1(iii)–Mn2(iv)–Mn3(iv)–Mn4(iv); associated ligands are Asp170, Glu354, Ala344, Asp342, Glu189, His332, Glu333, and four H_2_O molecules.^2,3^ The corresponding RESP charges are derived from B3LYP/6-31G*.^94,95^ The RESP fitting of the charges was performed using Multiwfn.^97^ The charge on backbone atoms of the coordinated residues on the OEC is carefully restrained on the link atoms, according to the standard residue charges of the original AMBER force field.^93^

All QM/MM calculations were performed using the multiscale module of the ORCA 5.0 suite, which incorporates the electrostatic embedding technique.^96,98,99^ The hydrogen link atom approach was employed to cut through C–C covalent bonds and the charge-shift (CS) scheme was used to avoid over polarization of the QM region. Along with the chlorin macrocycles, the axially coordinated ligands to Mg^2+^ were also treated at the QM level. For Chl_D1_ and Chl_D2_, the water molecule hydrogen bonded to the axially ligated water and ester group attached to the 13^2^-carbon position on ring E is also included in the QM region. Similarly, the axial histidines (His198 and His197) in case of P_D1_ and P_D2_ were also treated at the QM level. The phytyl chains were included in the QM region up to C^17^ (truncated as a methyl group) and the rest of the chain was treated in the MM region.

### 2.2. Geometry optimization

For geometry optimizations in the QM/MM framework, the complete system was further subdivided into two parts: active and static. The active region consists of atoms within the QM and MM regions that are flexible during the optimization, whereas the remaining MM atoms are fixed and only contribute to the electrostatics. The original pair-optimized QM geometries (*i.e.*, Chl_D1_–Pheo_D1_, Chl_D2_–Pheo_D2_ and P_D1_–P_D2_), for the ground state singlet states (*S* = 0) were used as starting structures.^24^ The ground triplet states (*S* = 1) of all RC pigments were optimized individually except for the central pair (P_D1_P_D2_), which is considered as a single dimeric unit. For individual pigments, complete amino acid residues and waters within 10 Å around the QM region were included in the active region, whereas a larger active region was chosen around the P_D1_P_D2_ pair (∼10 Å around each of P_D1_ and P_D2_). The Perdew–Burke–Ernzerhof (PBE) functional^100^ was used to optimize the QM regions using the def2-TZVP basis set,^101^ along with D3(BJ) dispersion corrections.^102,103^ Dense DFT integration grids (DefGrid2 in ORCA convention) were used in all optimizations. The resolution of identity approximation (RI) was used to speed up the calculation of Coulomb integrals with the corresponding auxiliary basis set (def2/J).^104,105^ All QM/MM geometry optimizations were performed using the L-BFGS optimizer.^106^

### 2.3. Excitation energies

Vertical triplet excitation energies were computed on the pair-optimized ground state singlet geometries (*i.e.* spin-restricted DFT reference), employing the Tamm-Dancoff approximation (TDA) to TD-DFT. It has been shown that the “triplet instability” problem of spuriously low-lying excitations for complex systems can be overcome to a large extent by using the TDA approach.^107^ In this work, we also considered the effect of this approximation on the singlet excitation energies of photosynthetic pigments, which remains a challenging problem for approximate TD-DFT.^108,109^ All TD-DFT calculations were performed using the range-separated ωB97X-D3(BJ) functional (modified version of ωB97X-V^110^ with D3BJ correction) along with def2-TZVP basis sets. The long-range-corrected functional has a fixed exact (Hartree–Fock) exchange of 16.7% (short-range) that increases to 100% at long range with a range-separation parameter of 0.30 a_0_^−1^. The performance of this functional towards the efficient treatment of excited states and electrochromic shifts using TD-DFT has already been confirmed in the past *via* direct comparisons with similarity transformed equation of motion coupled cluster theory (STEOM-CCSD).^84,111^ The RIJCOSX approximation^104^ was used to speed up the calculations and the corresponding auxiliary basis sets were used throughout. VeryTightSCF convergence criteria were applied throughout, along with dense integration grids (DefGrid2). The first 10 singlet (*S* = 0) and triplet (*S* = 1) excited states were computed for individual RC pigments as well as for oligomeric assemblies. This approach effectively describes the entire Q-band range and all low-lying excited states with local excitation (LE), charge-transfer (CT), and mixed LE/CT characters. The excited states for isolated pigments were computed using gas phase TD-DFT whereas in the case of the reaction center the electrostatic effects of the protein environment on the excited states were included through MM point charges of the entire PSII monomer. We further obtained the low-energy triplet (*S* = 1) excited states for pigment assemblies along the D1 [P_D1_P_D2_Chl_D1_Pheo_D1_] and D2 [P_D1_P_D2_Chl_D2_Pheo_D2_] branches (see Fig. 1). It is noted that specific pigment pairs at the RC are structurally uncoupled and that the geometries obtained by directly optimizing a tetramer are essentially identical compared to the combination of pairwise-optimized structures.^24^ The inclusion of tetramers in QM optimizations do not obviously alter the excited state energetics and provide the same qualitative picture of low-lying CT states and local excitons as the pair-optimized structures.

### 2.4. EPR parameters

The isotropic hyperfine coupling constants of all the hydrogen atoms are computed on the localized triplet states of individual pigments incorporating the effect of the protein *via* the QM/MM approach. All EPR parameters were computed within the framework of a DFT-based coupled-perturbed self-consistent field approach (CP-SCF)^112^ on the QM/MM optimized geometries of the triplet (*S* = 1) states with separate QM regions defined for each RC chromophore. For the hyperfine coupling constants and *g*-tensors, we used the TPSSh functional^113^ with Barone's EPR-II basis set^114^ on hydrogen atoms and def2-TZVP on the remaining atoms in the QM region. The RIJCOSX approximation and VeryTightSCF convergence criteria were used along with the highest DefGrid3 integration grids.^104^ The triplet *g*-tensors were computed in conjunction with the spin–orbit mean-field (SOMF) approximation for the spin–orbit coupling.^112,115^ The spin–spin contribution to the zero field splitting (ZFS) tensors^116^ (*D* and *E*) were computed using the restricted open shell Kohn–Sham (ROKS) framework, as this approach was shown to yield better agreement with experimental results than unrestricted (UKS) for triplet states of several organic molecules involving π electrons.^117^

## 3. Results and discussion

### 3.1. Singlet–triplet excitations in individual pigments

The electrostatic effects of the protein matrix are known to modulate the excited state properties of reaction center pigments.^84^ Previous work identified that the protein matrix is exclusively responsible for creating transverse and lateral excitonic asymmetry among the pigments within the PSII RC.^12,82,83^ This asymmetry leads to trapping of the excitation energy and initiation of primary charge-separation in the D1 branch. In the presence of the protein matrix the pigment with the lowest site energy is computed to be Chl_D1_.^12^ Detailed work on pigment assemblies additionally showed that the lowest singlet excited state is localized on the Chl_D1_–Pheo_D1_ pair and that this is usually a mixture of excitonic and charge-transfer (CT) Chl_D1_^*δ*+^Pheo_D1_^*δ*−^ character.^24^ The corresponding CT state involving the Chl_D2_–Pheo_D2_ pair on the inactive D2 is higher in energy, thus elucidating the excitonic asymmetry of the RC, where the protein matrix stabilizes excited CT states on the D1 branch. However, the explicit role of the protein electrostatics in controlling the excited state energetics of the triplet states has never been studied. It is also not clear if asymmetry exists at all in the case of triplet excitations. This information would be useful for understanding triplet-state formation and subsequently establish the role of protein matrix in photoprotection.

As a first step, we computed the singlet and triplet excitation energies of individual RC pigments using TD-DFT in the QM/MM framework. The Q and B bands of the absorption spectra of porphyrin-like macrocyclic compounds are described according to the Gouterman model,^118^ which involves excitations within the four frontier molecular orbitals HOMO−1, HOMO, LUMO, and LUMO+1, delocalized over the chlorin ring.^111^ For instance, the fundamental singlet excitation of the chlorophylls is the Q_*y*_ band (S_1_), corresponding to HOMO → LUMO and secondarily to HOMO−1 → LUMO+1 excitation. Based on the TD-DFT calculations, the lowest triplet excitations consist of two unpaired electrons, ferromagnetically coupled to each other in two singly occupied orbitals (SOMO 1, SOMO 2), also delocalized over the chlorophyll macrocycle.^119,120^ Our TDA-TDDFT results (see Tables S1–S5†) show that the two lowest energy triplet excited states (T_1_, T_2_) of RC chlorophylls are characterized by HOMO → LUMO (in the range of 1.22–1.30 eV) and HOMO−1 → LUMO (range of 1.73–1.78 eV) transitions. Furthermore, in all four central chlorophylls (*i.e.*, Chl_D1_, P_D1_, P_D2_, Chl_D2_) the two lowest triplet excited states (T_1_ and T_2_) are energetically lower than the corresponding singlet excitations (S_1_ and S_2_). This observation suggests that the lowest triplet local excitations are likely to result from spin–orbit induced inter system crossing (ISC) from the corresponding first singlet excited state (S_1_) of each chlorophyll.^84^

The computation of singlet excitation energies without protein electrostatics demonstrates that both Chl_D1_ and Chl_D2_ pigments have similar site energy in the gas phase (1.88 eV and 1.90 eV, respectively, see Table 1). Moreover, the nature of excitations and participating orbitals for the chlorophyll triplet remains consistent even in the absence of the explicit PSII protein environment. On the other hand, calculations done with full inclusion of protein electrostatics red-shifts the first excited state for both pigments. This effect is more pronounced for ^1^Chl_D1_ (1.82 eV) compared to ^1^Chl_D2_ (1.88 eV). Interestingly, similar spectral shifts are obtained for the lowest triplet state (T_1_), where we observed protein-induced red shifts highest for ^3^Chl_D1_ (70 meV) followed by ^3^Chl_D2_ (31 meV), ^3^P_D1_ (18 meV) and ^3^P_D2_ (23 meV). The excitation energy of ^3^Pheo_D1_ was found to be 17 meV higher than ^3^Chl_D1_, and about 10 meV higher than the T_1_ states of P_D1_, P_D2_ and Chl_D2_. Clearly, the signature of transverse excitonic asymmetry within the RC is preserved for the lowest localized triplet excitations. Nevertheless, it will be interesting to see how the absolute S_1_ and T_1_ excitation energies and S_1_–T_1_ gap are modulated by the protein matrix as these states should be involved in S–T intersystem crossing. The vertical excitation energies of the lowest singlet and triplet state along with the respective S–T gaps, in the presence and absence of the protein, are listed in Table 1. It is important to note that the protein matrix induces an asymmetry in tuning the S_1_–T_1_ gap for the accessory chlorophylls Chl_D1_ and Chl_D2_. In the case of Chl_D1_, both S_1_ and T_1_ are red-shifted by *ca.* 70 meV in the presence of the protein compared to the gas phase. In the case of Chl_D2_ the S_1_–T_1_ gap is 0.59 eV in the presence of the protein, similar to the gas phase (0.58 eV).

**Table 1:** Vertical excitation energies of the lowest singlet (S_1_) and triplet (T_1_) excited states along with the respective S–T gaps, in the presence and absence of the PSII protein matrix, calculated using TD-DFT (ωB97X-D3BJ/def2-TZVP). ΔT_1_ represents the geometry relaxation of the first triplet state. The gas-phase excited state calculations were performed using the QM/MM optimized geometries. All values are reported in eV

Method	Δ*E*		TD-DFT (in protein)			TD-DFT (gas-phase)		
RC pigment	T_1_–S_0_ (opt)	ΔT_1_ (opt)	S_1_	T_1_	S_1_–T_1_	S_1_	T_1_	S_1_–T_1_
Chl_D1_	0.920	0.300	1.818	1.220	0.598	1.884	1.290	0.594
P_D1_	0.994	0.311	1.859	1.305	0.554	1.898	1.323	0.575
P_D2_	0.978	0.313	1.859	1.291	0.568	1.897	1.314	0.583
Chl_D2_	0.970	0.318	1.878	1.288	0.590	1.900	1.319	0.581

**Fig. 3:**
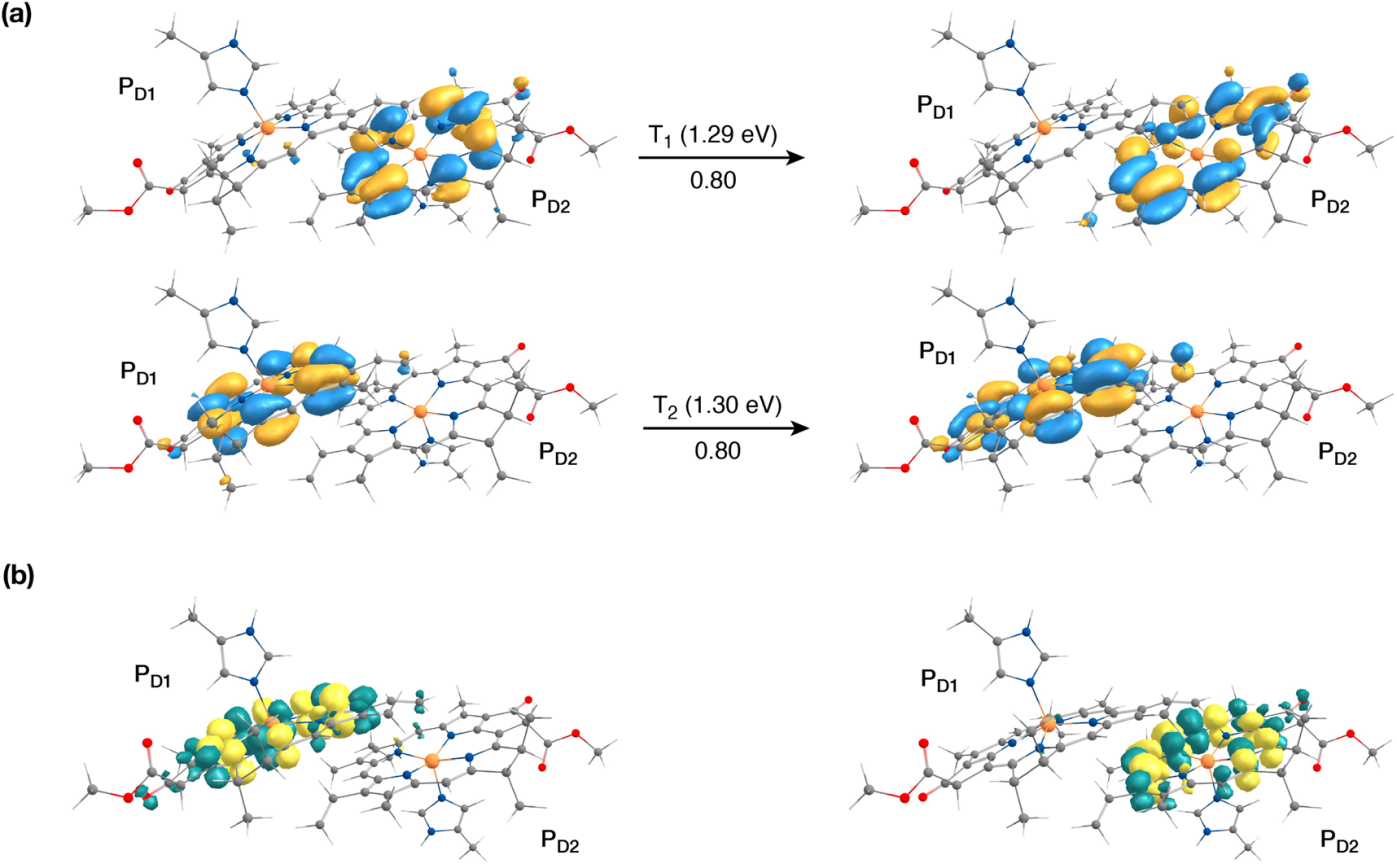
The nature of the lowest triplet excitations shown for the P_D1_P_D2_ pair: (a) donor and acceptor natural transition orbitals and relative contributions of the transition to the given excited state (here the NTOs coincide with the HOMO and LUMO orbitals of the individual pigments); (b) the corresponding difference densities for the lowest singlet-triplet excitations on P_D1_ and P_D2_.

The singlet excited states on the central pair P_D1_P_D2_ in the presence of the protein point charges show that the lowest singlet excited states at 1.86 eV and 1.88 eV are a superposition of local excitons on P_D1_ and P_D2_, respectively (Table S6†). The lowest CT state involving the central pair (P_D1_^*δ*+^P_D2_^*δ*−^) is significantly higher (*ca.* 3.2 eV) than the S_0_. On the other hand, in the case of triplet excitations, the two lowest triplet states are isoenergetic and correspond to triplet excitons localized on P_D2_ (T_1_, 1.29 eV) and P_D1_ (T_2_, 1.30 eV) respectively (see Fig. 3a and b). Our results do not show any low-energy triplet state of the same character as the ^1^[P_D1_^*δ*+^P_D2_^*δ*−^] CT state mentioned above. Moreover, each triplet excitation spanning a range of 1.40–1.50 eV is attributed to individual pigments (see Table S6†), suggesting that the triplet excitons are entirely localized on either of the two chlorophyll molecules (P_D1_ or P_D2_) and therefore there is no superposition, in contrast to the singlet excitons. The absence of a low-lying triplet state with CT character is also indicative of the fact that a radical-pair charge recombination may not be favorable to form ^3^[P_D1_P_D2_] states in the RC. However, it cannot be excluded that delocalized triplet excited states exist at higher energies, similar to the singlet CT excitations.^12,24^

### 3.2. Singlet–triplet excitations in pigment assemblies

In photosynthetic RCs the excitation profiles of individual pigments are far from complete, and a thorough understanding of the initial charge-separation and charge recombination events requires insights from excitation energetics of multiple pigments. For instance, ^1^[P_D1_^*δ*+^Pheo_D1_^*δ*−^] and ^1^[Chl_D1_^*δ*+^Pheo_D1_^*δ*−^] charge-transfer (CT) excitations were found significantly stabilized, lower than the local excitons, due to the differential effect of the protein matrix.^18,24^ Moreover, the lowest (Q_*y*_) excitation of Chl_D1_ was found to be mixed significantly with the ^1^[Chl_D1_^*δ*+^Pheo_D1_^*δ*−^] CT state.^18^ A number of experimental studies suggest that based on the characteristic spin polarization pattern of the EPR spectra, the observable triplet state should be formed from a charge recombination of the primary radical pair.^37,56,58,61,64,66^ This further necessitates a quantitative description of the excitation profiles of groups of pigment assemblies, in order to establish a connection between the singlet-triplet CT excitations and the experimentally observable triplet state. Towards this objective, we first computed the low energy singlet and triplet excited states for the tetrameric pigment assemblies along the D1 [P_D1_P_D2_Chl_D1_Pheo_D1_] and D2 [P_D1_P_D2_Chl_D2_Pheo_D2_] branches.

The most common mechanism of triplet formation in organic chromophores involves a spin–orbit-induced intersystem crossing (ISC) but singlet fission, radical pair ISC, or spin–orbit charge-transfer ISC can result in triplet formation, particularly in systems with donor–acceptor pigment pairs.^54,89,121,122^ Similar studies on biomimetic assemblies have reported that low-lying CT states can promote triplet formation through a charge recombination of donor–acceptor radical pairs followed by ISC.^123,124^ Our TD-DFT results show that the lowest singlet excitations in the [P_D1_P_D2_Chl_D1_Pheo_D1_] branch correspond to ^1^[P_D1_^*δ*+^Pheo_D1_^*δ*−^] (1.548 eV) and ^1^[Chl_D1_^*δ*+^Pheo_D1_^*δ*−^] (1.693 eV) CT states, respectively (Table 2). These results are further in line with recent QM/MM and TDDFT studies.^18,24^

**Table 2:** Excited state properties of the D1 tetramer (P_D1_P_D2_Chl_D1_Pheo_D1_) computed using (TDA)-TDDFT with QM/MM at the ωB97X-D3BJ/def2-TZVP level of theory. The nature of excited states is labelled as local excitons (LE) or charge-transfer (CT), based on natural transition orbitals (NTOs) for singlet states and canonical molecular orbitals for the triplet states. *E*_S_ and *E*_T_ represent the singlet and triplet vertical excitation energies (VEE) in eV; *f*_osc_ are the corresponding oscillator strengths

Roots	*E*_S_	*f*_osc_	Transition	*E*_T_	Transition
1	1.548	0.00	CT (P_D1_ →Pheo_D1_)	1.215	LE (Chl_D1_)
2	1.693	0.06	CT (Chl_D1_ → Pheo_D1_)	1.291	LE (P_D2_)
3	1.801	0.32	LE (Chl_D1_)	1.303	LE (P_D1_)
4	1.807	0.02	CT (P_D2_ → Pheo_D1_)	1.386	LE (Pheo_D1_)
5	1.855	0.39	LE (P_D1_) + LE (P_D2_)	1.548	CT (P_D1_ → Pheo_D1_)
6	1.882	0.05	LE (P_D1_) + LE (P_D2_)	1.681	LE (Pheo_D1_)
7	2.023	0.00	CT (P_D1_ → Pheo_D1_)	1.708	CT (Chl_D1_ → Pheo_D1_)
8	2.033	0.17	LE (Pheo_D1_)	1.731	LE (Chl_D1_)
9	2.251	0.00	CT (Chl_D1_ → Pheo_D1_)	1.773	LE (P_D2_)
10	2.340	0.00	CT (P_D2_ → Pheo_D1_)	1.778	LE (P_D1_)
11	2.385	0.04	LE (Chl_D1_)	1.807	CT (P_D2_ → Pheo_D1_)
12	2.409	0.03	LE (P_D2_)	2.023	CT (P_D1_ → Pheo_D1_)

**Fig. 4:**
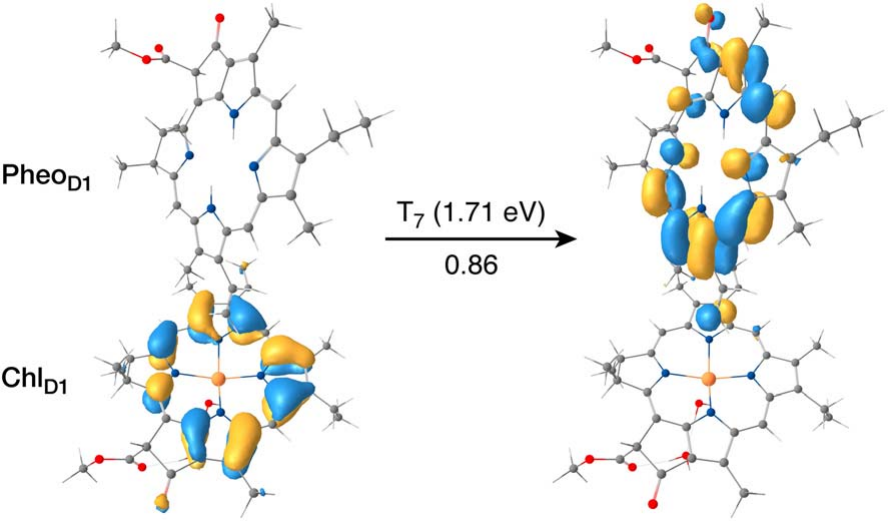
The identity of the triplet excited state with significant [Chl_D1_^*δ*+^Pheo_D1_^*δ*−^] charge transfer character in terms of canonical molecular orbitals and their contribution to the given excitation (calculation performed on the [P_D1_P_D2_Chl_D1_Pheo_D1_] tetramer, but only the implicated pigment pair is depicted).

**Fig. 5:**
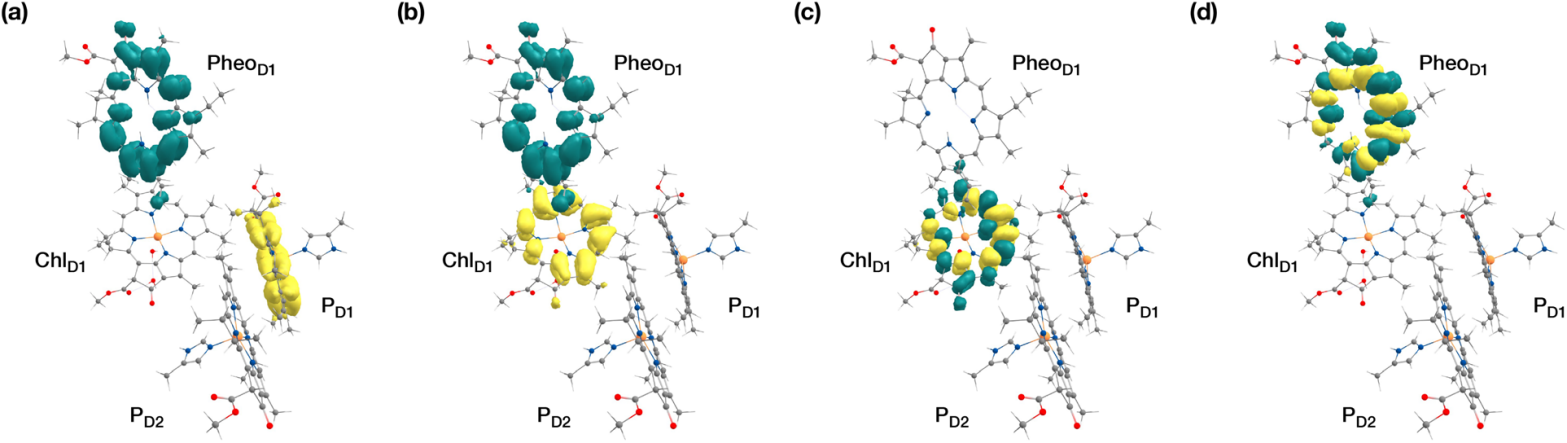
Difference densities describing the lowest singlet-triplet excitations of the D1 branch in PSII: (a) the lowest triplet excitation with ^3^[P_D1_^*δ*+^Pheo_D1_^*δ*−^] charge transfer character; (b) the lowest triplet excitation with ^3^[Chl_D1_^*δ*+^Pheo_D1_^*δ*−^] charge transfer character; (c) local ^3^Chl_D1_ excitation; (d) local ^3^Pheo_D1_ excitation.

The results presented and analyzed in terms of natural transition orbital (NTO) compositions (see Table 2) and (TDA)-TDDFT difference densities show that the lowest triplet excited state of the D1 tetramer (T_1_ at 1.215 eV) is fully localized on Chl_D1_, which also exhibits the lowest site energy (S_1_ at 1.801 eV) among all RC pigments. The second and third triplet states (T_2_ at 1.291 eV and T_3_ at 1.303 eV) are localized excitations on P_D2_ and P_D1_ respectively. These results are in line with those obtained for the pigment monomers and dimers. Most importantly, we identified the “spin-flipped” triplet states ^3^[P_D1_^*δ*+^Pheo_D1_^*δ*−^] (1.548 eV) and ^3^[Chl_D1_^*δ*+^Pheo_D1_^*δ*−^] (1.708 eV, Fig. 4) that are isoenergetic with the lowest singlet CT states (see Table 2). The corresponding TD-DFT difference densities for the low-energy CT triplet excitations ^3^[P_D1_^*δ*+^Pheo_D1_^*δ*−^] and ^3^[Chl_D1_^*δ*+^Pheo_D1_^*δ*−^] are depicted in Fig. 5. It is noteworthy that all the RC pigments exhibit a triplet exciton lower than the above donor–acceptor CT states.

**Fig. 6:**
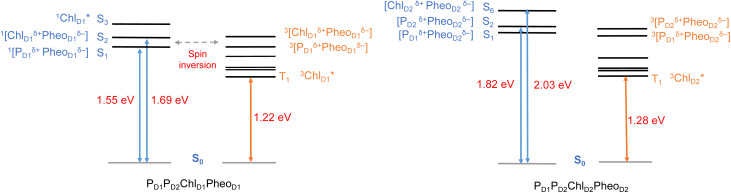
Schematic representation of selected low-energy singlet and triplet excitations for the P_D1_P_D2_Chl_D1_Pheo_D1_ and P_D1_P_D2_Chl_D2_Pheo_D2_ tetrameric assemblies computed using (TDA)-TDDFT with QM/MM at the ωB97X-D3BJ/def2-TZVP level of theory.

All the low-energy triplet states are dominated by local excitations on Chl_D1_, P_D1_, P_D2_ and Pheo_D1_, all lower in energy than the lowest triplet CT states. This is in contrast to singlet excitations wherein the low-energy profile is dominated by mixed local excitons and CT excitations or states with pure CT character. Furthermore, most local excitons are blue-shifted compared to the donor–acceptor CT states. Overall, our results clearly demonstrate that low-energy singlet and triplet excited state manifolds differ significantly for primary donor–acceptor pairs in the RC. A detailed schematic representation comparing the complete low-energy spectrum (singlet and triplet excitations) of the RC is provided in Fig. 6. Based on our calculations one would expect that the observable triplet state in the RC can be formed from recombination of either of these radical pairs that subsequently decays to the neutral ground-state chlorophyll triplet ^3^Chl_D1_. This mechanism is different from the formation of other triplet states (*e.g.* in light-harvesting antennae) where ^3^Chl formation is mediated by triplet–triplet energy transfer (T-TET)^54,55,70^ or direct intersystem crossing from a singlet excited state.^54^ The singlet-triplet excitation spectra of the D2 tetramer [P_D1_P_D2_Chl_D2_Pheo_D2_] (see Fig. S1 and Table S7†) are also comprised of CT triplet excitations corresponding to ^3^[P_D2_^*δ*+^Pheo_D2_^*δ*−^] (1.706 eV), ^3^[P_D2_^*δ*+^Pheo_D2_^*δ*−^] (1.816 eV) and ^3^[Chl_D2_^*δ*+^Pheo_D2_^*δ*−^] (2.032 eV) respectively. The lowest triplet exciton in the D2 side is localized on Chl_D2_ at 1.279 eV.

### 3.3. Relaxed triplet states

In the previous section we explored the influence of the protein matrix on the excitonic asymmetry for singlets and triplets, where the lowest energy excitons were found to be localized on Chl_D1_. Interestingly, while singlet excitation energy transfer (EET) within the RC seems unlikely due to rapid charge separation, the protein matrix tends to delocalize triplet states over the four chlorophyll pigments. Understanding this phenomenon of triplet delocalization among RC pigments is crucial for comprehending the mechanisms of photo-quenching and photoprotection in PSII.^51,87^ Moreover, obtaining accurate estimates of the triplet energy gaps among individual pigments is necessary to determine the actual rates of photo-quenching. To address this, we conducted further QM/MM geometry optimizations of the individual pigments (Chl_D1_, Chl_D2_, P_D1_, and P_D2_) in their singlet and triplet states, enabling us to estimate the adiabatic T_1_–S_0_ energy gaps for each chlorophyll.

Previous site-directed mutagenesis experiments on D1-H198G, combined with low-temperature optical difference spectroscopy, conducted by Diner *et al.*,^9^ reported shifts in the difference spectra of P_D1_^+^/P_D1_ and Y_*Z*_˙/Y_*Z*_, as well as displacements in the midpoint potential of P_D1_^+^/P_D1_. However, the mutation had no effect on the difference spectra or EPR properties corresponding to ^3^P_680_. Schlodder *et al.*^125^ performed similar studies on D1-T179H mutants, which involve the ligand H-bonded to the axially bound water of Chl_D1_, and observed shifts in the Q_*y*_ band and EPR signals upon triplet formation. The T–S absorption spectra of photosynthetic pigments in D_1_D_2_Cyt*b*559 complexes were also investigated by Renger *et al.*,^15,42^ and more recent phosphorescence measurements^73,74^ supported the notion that the triplet state is localized on an RC chlorophyll different from the one accommodating the stable positive charge. FTIR measurements indicated that the triplet is localized on a chlorophyll distinct from the primary cation-stabilizing chlorophyll, based on the vibrational peak of the 13^1^-keto C=O keto arising from differences in H-bonding interactions.^20^ These experimental observations, combined with the latest experimental and theoretical descriptions of the primary events at the RC of PSII that identify Chl_D1_ as the primary donor, consistently support the idea that the accessory chlorophyll Chl_D1_ is the site of the most stable triplet state.

Here, we determined the TD-DFT vertical excitation energies for ^3^[P_D1_P_D2_] and ^3^Chl_D2_ to be 1.29 eV and 1.28 eV, respectively (see Table 1). Consequently, the lowest energy triplet excitation was found to be localized on Chl_D1_, consistent with the above findings. Additionally, we observed that the QM/MM geometry relaxation had a similar effect of approximately 0.3 eV on the triplet state for each chlorophyll in the reaction center (Table 1). The EPR/ENDOR and FTIR spectra obtained from temperature-dependent studies estimated energy differences between ^3^Chl_D1_ and ^3^P_D1_ of 8–13 meV from isolated RCs and 11 meV from core complexes.^9,62,74,126^ Our computational results align with these experimental observations, indicating that the triplet state on Chl_D1_ is also the lowest in energy among all pigments at the reaction center.^15,42^ However, given the close spacing of energy levels, it is expected that at higher temperatures, an equilibrium would exist among the triplet states of P_D1_, P_D2_, Chl_D1_, and Chl_D2_, resulting in the delocalization of the observable triplet state over more than one chlorophyll molecule. These conclusions are consistent with recent FTIR studies conducted by Noguchi and co-workers.^87^ Therefore, our findings support both the localization of the triplet on the specific chlorophyll center (Chl_D1_) at low temperatures and the decrease in triplet intensities due to delocalization at ambient temperatures.

### 3.4. EPR parameters of triplet chlorophylls

Magnetic resonance studies coupled with photoexcitation, especially time-resolved electron paramagnetic resonance (EPR) spectroscopy, have been widely applied to characterize the triplet states and organic radicals involving photosynthetic pigments.^36,38,40,54,55,71,75,76,127^ The triplet states involving RC, antenna chlorophylls as well as carotenoids have been characterized using transient and pulse ENDOR spectroscopy,^37,39,66,126,128,129^ however a number of these studies led to varying observations depending on the type of preparation and conditions used, as in the case of D_1_D_2_Cyt*b*_559_ particles or PSII core complexes. DFT methods have also been used to quantify EPR properties of photosynthetic pigments but they have excluded so far the effect of protein electrostatics.^127,128^ Therefore, in order to obtain reliable quantitative insights regarding the influence of the local protein environment on the localisation site of the triplet states, here we compute for the first time the EPR properties of each RC pigment in their triplet (*S* = 1) geometries using the present QM/MM setup.

The accurate determination of zero field splitting (ZFS) parameters *D* and *E* is important to characterize the spatial extent and specific location of the triplet-state spin densities. From a methodological perspective, the accuracy of the spin–spin contribution of the *D*-tensors (*D*_ss_) for organic radicals is significantly affected by spin contamination, and ROKS approaches show better performance than UKS approaches for predicting the correct sign and the ZFS tensor orientation in organic triplets.^117^ Based on our calculations (see Table S9†) we observe good agreement despite a small systematic underestimation of the magnitude of the ZFS for the RC triplets, as also reported in the past for isolated Chl *a* triplets.^117^ Our calculations nevertheless confirm that the lowest triplet state is localized on a monomeric chlorophyll at the RC, as can be concluded from the corresponding ZFS parameters and comparison with those of isolated Chl *a*. This appears to rule out the possibility that the observed triplet is delocalized at low temperatures. From the first series of EPR studies on chlorophyll triplets in photosynthetic RCs, Rutherford *et al.*^56,61^ and Van Mieghem *et al.*^58^ proposed that the observable triplet is localized on a pigment whose ring plane is tilted at an angle of 30° with respect to the membrane plane. Following on the 1.9 Å crystal structure of PSII,^8^ this was assumed to be either of the accessory chlorophylls, Chl_D1_ or Chl_D2_. Based on our QM/MM model and EPR calculations, we estimated an angle of about 37° between the chlorophyll plane and the approximate membrane plane, the *z*-axis of the ZFS tensor and the molecular *z*-axis (perpendicular to the porphyrin ring plane) being approximately collinear. However, one still cannot assign the triplet state of the RC to a specific pigment only based on the ZFS parameters.

A more sensitive tool that offer insights into the electronic nature of the triplet states is the electron-nuclear hyperfine coupling (HFC) for protons and heavier nuclei strongly interacting with it. We computed the ^1^H HFC constants for each of the chlorophyll triplet states explicitly accounting for the protein electrostatics. From our calculations, we can assign the EPR coupling constants to each proton corresponding to the chlorophyll triplet state (Table 3). It has been argued based on experiments that ^3^P_680_ is localized on Chl_D1_ or Chl_D2_, based on the low number of contacts of the three methyl groups (2, 7 and 12). We also conclude that the peak corresponding to the highest positive HFC should be assigned to the freely rotating methyl group at position 12, followed by that of 2, and this is consistent for all the RC pigments. Our assignment of the hyperfine coupling constants is also consistent with DFT computed Mulliken spin populations of the neighboring carbon atoms of the chlorin macrocycle (see Fig. S2†). Overall, C^12^ has the highest spin population (0.293 in Chl_D1_) in the chlorin ring, which consequently leads to a large proton hyperfine coupling in the C^12^ methyl protons. The spin population at C^2^ and C^7^ are comparatively lower. The assignment of the HFC constants at position 2 is also interesting, because the signal corresponding to these protons is not clearly assigned in ENDOR studies of isolated RC (D_1_D_2_Cyt*b*_559_) samples.^39^ Interestingly, the largest contribution for each chlorophyll is seen to arise for the methyl protons oriented towards the perpendicular *z*-axis of the molecule. The negative values of the HFCs are assigned to the methine (CH) protons on the plane of the chlorin macrocycle (5, 10 and 20) because their isotropic couplings arise from spin polarization effects. Among these methine (CH) protons the carbon with highest spin density leads to more a negative value of ^1^H HFC due to a higher spin polarization and this trend is consistent among all the four RC pigments. In the recent work by Niklas and coworkers,^37^ the hyperfine coupling constants for the protons at C^17^ and C^18^ were not clearly determined for ^3^P_680_. From our calculations, we observe that for all the chlorophylls the proton at position 18 has a higher isotropic ^1^H HFC than position 17. Also, the corresponding spin population analysis of the macrocyclic carbon atoms indicate a higher spin density at C^19^ than C^16^. This trend is also consistent among all the RC chlorophylls (Chl_D1_, Chl_D2_, P_D1_ and P_D2_), and therefore our QM/MM calculations indicate the experimentally observed HFC of 2.99 in ^3^P_680_ likely arises from position 18.

**Table 3:** Experimental and calculated hyperfine coupling constants (in MHz) for ^3^P_680_, other ^3^Chl *a* species, and triplet states of the pigments in PSII RC, computed with the TPSSh functional, the EPR-II basis set on H atoms and the def2-TZVP basis set on other atoms

	Triplet state	10 (CH)	20 (CH)	5 (CH)	7 (CH_3_)	12 (CH_3_)	2 (CH_3_)	18 (CH)	17 (CH)	3′ (CH)	3′′ (CH_2_)
ENDOR^37,39,127^	^3^P_680_	−10.03	−7.88	−4.79	0.62	10.35	4.80	2.99	n.d.	0.91	−1.30
	^3^Chl *a* (WSCP)	−10.20	−7.70	−5.70	1.10	10.70	4.70	2.60	n.d.		
	^3^Chl *a* (MTHF)	−11.44	−7.20	−6.20	n.d.	7.40	n.d.				
DFT	^3^Chl *a* (gas-phase)	−5.12	−5.21	−3.32	0.97	10.77	5.61	4.81	3.96	0.69	−1.64
	^3^Chl *a* (MTHF)	−7.20	−7.32	−4.96	0.39	10.61	5.69	3.14	2.46	0.16	−1.65
	^3^Chl_D1_ (gas-phase)	−6.63	−6.77	−5.61	0.61	10.95	5.35	2.90	1.78	0.39	−2.04
QM/MM	^3^Chl_D1_	−6.98	−6.18	−5.64	1.25	12.27	5.68	2.59	1.28	0.52	−2.80
	^3^Chl_D2_	−7.41	−6.42	−5.63	0.71	12.41	5.31	3.06	1.58	0.64	−2.19
	^3^P_D1_	−6.02	−5.17	−5.59	1.05	10.86	4.78	2.47	1.19	0.58	−2.42
	^3^P_D2_	−1.82	−4.13	−1.08	1.07	11.67	5.93	4.18	4.69	3.59	−1.76

We have also identified contributions from the vinyl group (3′, 3′′), the peaks of which were not clearly assigned in previous spectroscopic studies. The negative HFC at 3′′ is likely due to spin polarization from C^3′′^, and the magnitude is consistent with the corresponding spin populations. However, the orientation of the vinyl group of P_D2_ is particularly noteworthy here. It is known that in P_D2_ the vinyl CH_2_ is slightly out of plane from the chlorin macrocycle, and our results indicate that this significantly affects the spin density distribution of the vinyl carbons. This clearly explains why the ^1^H HFC of the vinyl protons in P_D2_ differ significantly from the other RC chlorophylls.

**Fig. 7:**
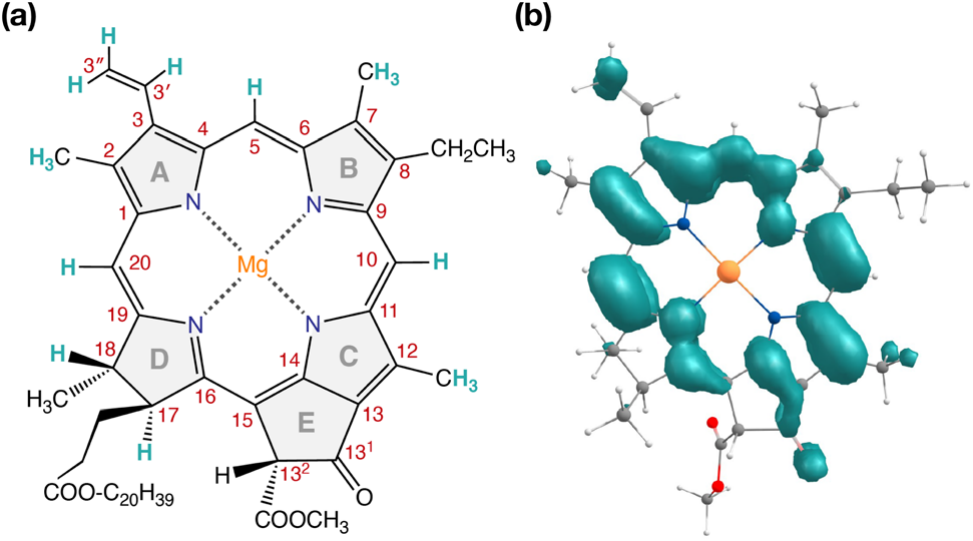
(a) Structure of Chl *a* with carbon atom numbering and spectroscopically important hydrogen positions indicated. (b) Computed spin density distribution of triplet (*S* = 1) Chl *a*.

Our QM/MM methodology therefore not only reproduces the experimental EPR/ENDOR results obtained from intact PSII core samples but also accounts for local perturbations that might affect EPR signals from isolated RC samples. Overall, the triplet spin distribution of individual chlorophylls (Fig. 7b) remain unchanged for isolated RC samples.^87^ The EPR parameters however, are not sufficiently sensitive to the protein environment to enable confident differentiation between the chlorophylls of the RC and it is not possible to assign the spectroscopically observable triplet state to a single RC chlorophyll based on EPR parameters alone. Nevertheless, the lowest triplet excitations and the energetically most stable triplet state are found on Chl_D1_ and, hence, the combined results of all our calculations show a clear preference to assign this state to a triplet state localized on the accessory chlorophyll Chl_D1_.

### 3.5. Electrostatic effects by plastoquinone Q_A_ and the OEC

Until now, we discussed the optical properties of primary electron transfer processes in PSII, where the OEC is in its dark-stable state (S_1_) and Q_A_ is a neutral plastoquinone. Under normal conditions, the charge recombination of the primary charge separated states [Chl_D1_^+^Pheo_D1_^−^]^4,11,16^ (or [P_D1_^+^Pheo_D1_^−^])^24^ is prevented by forward electron transfer from Pheo_D1_ to Q_A_ (within a few hundred ps). The oxidation of Y_*Z*_ (the redox-active tyrosine residue that interfaces the OEC with the RC) by P_D1_^+^ occurs instead within 25 ns to 50 μs.^5^ Both processes contribute to formation and modulation of an electrostatic gradient across the transmembrane region, which, coupled with intrinsic protein matrix effects, tunes the thermodynamics and kinetics of electron transfer pathways. Based on reported timescales, the oxidation of OEC by the Y_*Z*_ (50 μs to 4 ms), and electron transfer from Q_A_ to Q_B_ (0.2–0.8 ms) are the two main rate-limiting steps in PSII. These electron transfer processes thus eventually create the next stable intermediate of the RC with an oxidized OEC and reduced Q_A_ (S_2_Q_A_^−^)_._ On the other hand, in extreme conditions such as prolonged light exposure the plastoquinone (PQ) pool in thylakoid membranes can remain reduced, abolishing electron transfer from Q_A_ to Q_B_ and allowing Q_A_^−^ to accumulate. This can further drive competing secondary electron transfer pathways leading to triplet formation in the RC.

**Fig. 8:**
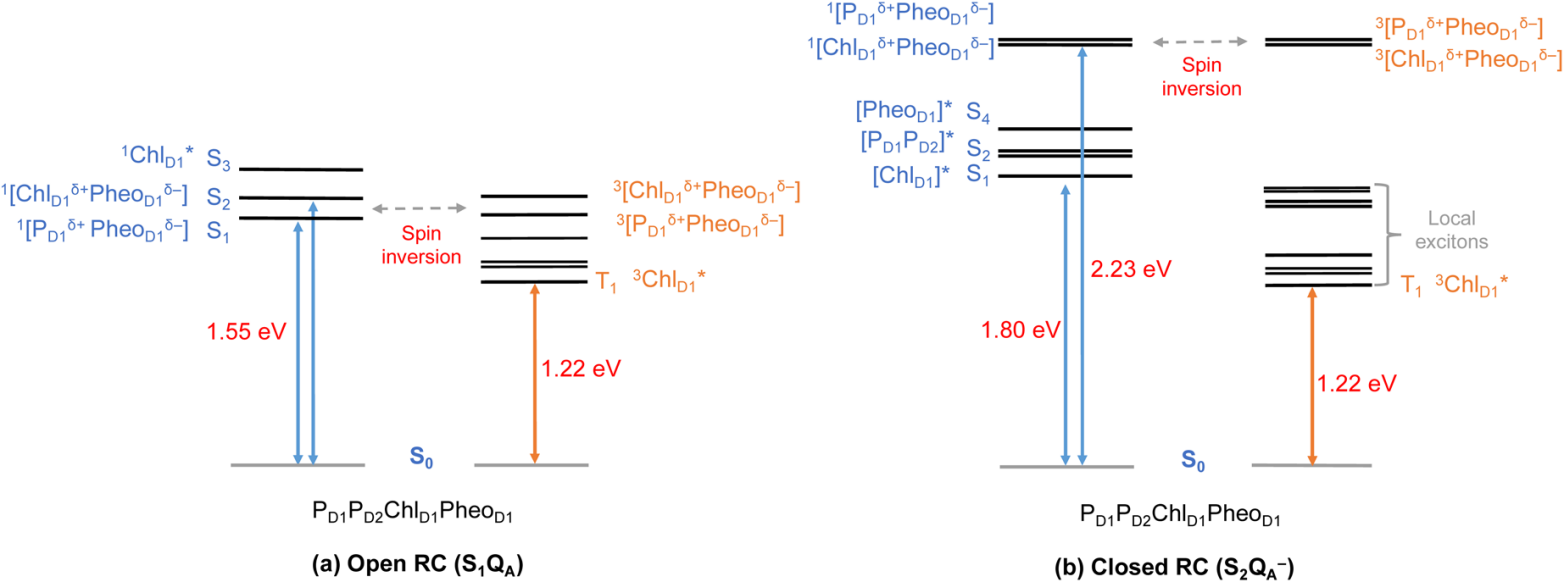
Schematic representation of the singlet and triplet vertical excitation energies of the P_D1_P_D2_Chl_D1_Pheo_D1_ tetramer in: (a) the open RC (S_1_Q_A_), and (b) the closed RC (S_2_Q_A_^−^). All energies are reported relative to the ground state singlet (S_0_).

In view of the above, as a next step we performed TD-DFT calculations on the “closed” RC, where the OEC is modelled in the S_2_ state of the Kok–Joliot cycle and Q_A_ is reduced, *i.e.*, the S_2_Q_A_^−^ state. Our excited state calculations on the [P_D1_P_D2_Chl_D1_Pheo_D1_] assembly (Table S8†) reveal interesting results. The low-energy spectrum (see Fig. 8) in the presence of the semiquinone Q_A_^−^ is dominated by local excitations both for singlets and triplets, in stark contrast to the case when Q_A_ is neutral and available to accept electrons. The relative stability of site energies (Chl_D1_, P_D1_, P_D2_ and Pheo_D1_) also explains the longer lifetime of chlorophyll excited states and high fluorescence yields observed in closed RCs.^26,45^ Moreover, the ^1^[Chl_D1_^*δ*+^Pheo_D1_^*δ*−^] CT state is 2.23 eV higher than the ground state and thus considerably blue-shifted compared to the open RC (1.69 eV). This is in line with previous experimental hypotheses regarding reduced charge separation due to the electrostatic repulsion of Q_A_^−^.^26,32,79^ Interestingly, we also find that the two low-energy CT states ^1^[Chl_D1_^*δ*+^Pheo_D1_^*δ*−^] (2.231 eV) and ^1^[P_D1_^*δ*+^Pheo_D1_^*δ*−^] (2.276 eV) are almost isoenergetic for the closed RC (Table S8†). This is clearly an effect of the differential influence of oxidized OEC and Q_A_^−^ on the primary donor–acceptor pairs, with Pheo_D1_^−^ and P_D1_^+^ being more destabilized than Chl_D1_^+^ due to their spatial proximity to Q_A_^−^ and/or the oxidized OEC respectively (Fig. S4;† Pheo_D1_ is the closest pigment to Q_A_ with an edge-to-edge distance of 8.8 Å, and a center-to-center distance of 13.2 Å, while P_D1_ is closest to OEC with a distance of about 17.2 Å).

Studies on charge recombination reactions have shown that formation of RC triplet states can be influenced not only by the presence of the semiquinone (Q_A_^−^) but also by the complete absence of Q_A_ (*e.g.*, isolated D_1_D_2_Cyt*b*_559_ samples) or the double reduction of Q_A_.^45,46^ In some experiments conducted at cryogenic temperatures the spin-polarized triplet state was only detected when Q_A_ was doubly reduced (Q_A_^2−^ or Q_A_H_2_) and not when it was singly reduced, which led to controversies about whether or not primary charge separation can occur in the presence of Q_A_^−^. Studies that monitored the light-induced triplet signals with different redox states of Q_A_ using EPR spectroscopy, reported higher triplet yields but shorter life times (*t*_1/2_ < 20 μs) with Q_A_^−^ (closed RC).^29,32,33^ On the other hand, Feikema *et al.* based on time-resolved EPR measurements on PSII core samples reported that the yield of the triplet state with a singly reduced Q_A_^−^ did not differ significantly from those with Q_A_H_2_.^29^ In the case of Q_A_H_2_ however, the chlorophyll triplet was reported to have a much extended lifetime (*t*_1/2_ ∼ 1–2 ms) and this has been attributed to the absence of Q_A_^−^ to quench chlorophyll triplet states in PSII. Moreover, flash-induced PSII activity measurements showed the extent of D1-photodamage due to ^1^O_2_ to be most pronounced in the S_2_ and S_3_ states of the OEC, and this also has been correlated to other competing back reactions.^47,50^ Hence, the pathway of triplet formation and the dependence of the singlet–triplet excitations on the redox state of the Q_A_ and OEC remain unclear, yet they are crucial to understand both the control of primary processes by the transmembrane electrostatic gradient and the photoprotection mechanisms of PSII.

**Fig. 9:**
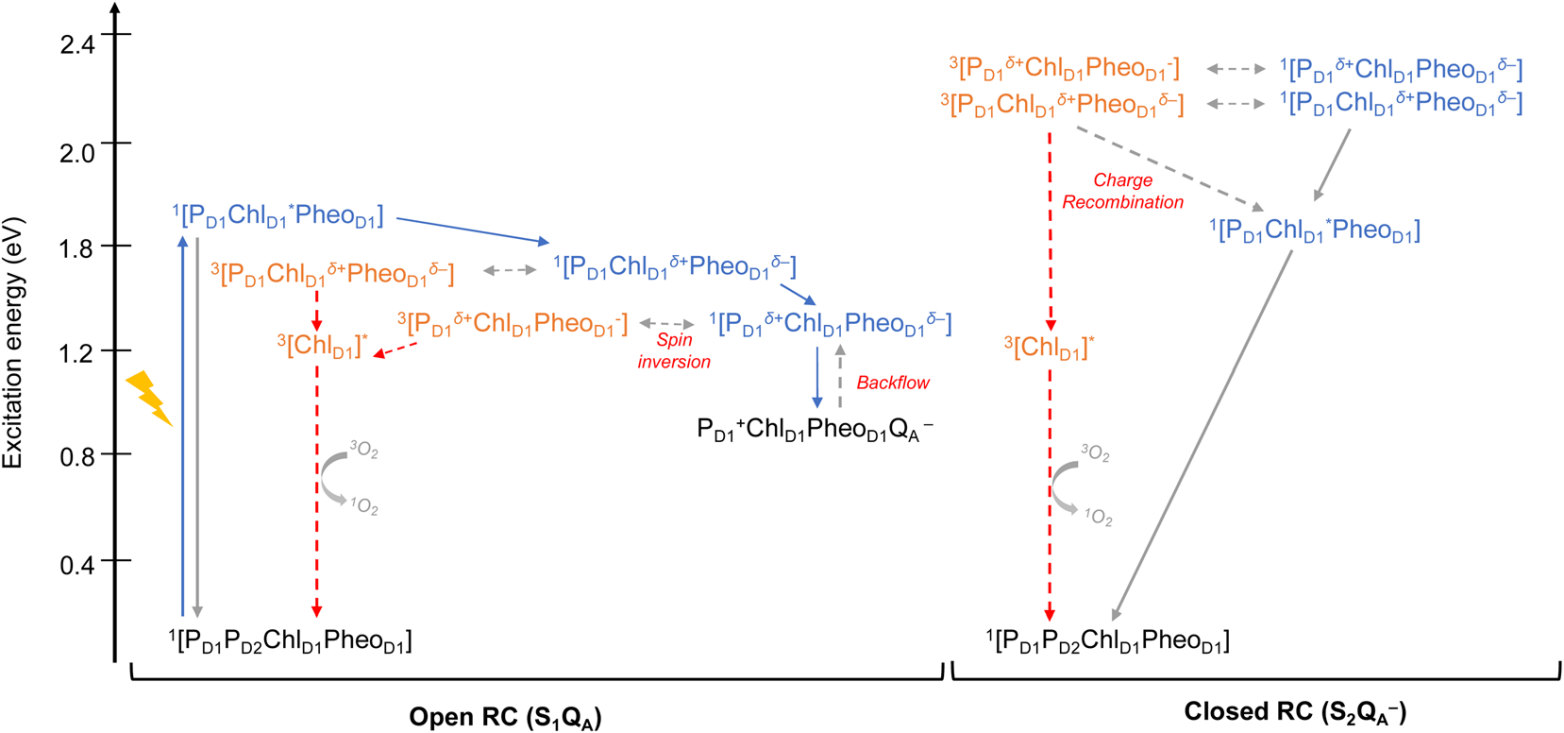
Schematic representation of singlet and triplet excitation energies, charge transfer pathways, charge recombination and triplet forming routes in the PSII-RC (a) OEC in S_1_, Q_A_ is neutral (b) OEC in S_2_, Q_A_ is singly-reduced. All energies are computed in eV relative to the ground state singlet (S_0_). Singlet excited states are shown in blue, triplet states in orange, forward electron transfer with blue arrows, singlet/radiative decay routes with grey solid arrows, backflow/spin inversion/charge recombination with grey dotted arrows, triplet routes in red.

As seen from the excitation energy profiles (Fig. 8), the energetics of the singlet and triplet charge transfer excitations can be directly influenced by the redox state of surrounding cofactors, particularly Q_A_. A more comprehensive overview of the singlet and triplet excitation energies, charge transfer pathways, charge recombination and triplet forming routes, is provided in Fig. 9. Based on our results, it can be suggested that formation of triplet states at the RC should be preceded by charge recombination of the primary radical pair [Chl_D1_^+^Pheo_D1_^−^] or [P_D1_^+^Pheo_D1_^−^] formed from the corresponding CT states. Subsequently, a very important aspect when discussing molecular mechanisms of photoprotection involves the acceptor side of PSII. Pheo_D1_ is the site of the primary anion radical Pheo_D1_^−^, following charge separation.^4,11,12,15,79^ In normal physiological conditions the electron is rapidly transferred to Q_A_ (Pheo_D1_^−^Q_A_ → Pheo_D1_Q_A_^−^). The thermodynamic driving force for this step is governed by the relative midpoint potentials of Pheo_D1_/Pheo_D1_^−^ and Q_A_/Q_A_^−^ and is controlled by local pigment–protein interactions.^32^ However, the reduction of Q_A_ to Q_A_^−^ can lead to the following alternate possibilities: (a) direct charge recombination with P_680_^+^ to ^1^P_680_^*^ and finally the ground state, (b) backward electron transfer onto Pheo_D1_ to form ^1^[P_680_^+^Pheo_D1_^−^] or (c) formation of the charge recombination triplet ^3^[P_680_^+^Pheo_D1_^−^] which finally localizes on Chl_D1_*i.e.*, the triplet route. Calculation of the Pheo_D1_ electron affinity suggests that Pheo_D1_^−^ formation is disfavored by *ca.* 0.5–1 eV in the presence of a reduced Q_A_^−^. The electrostatic repulsion of Q_A_^−^ destabilizes the primary radical pair [P_680_^+^Pheo_D1_^−^], but also inhibits forward electron transfer. This might cause spin inversion from ^1^[P_680_^+^Pheo_D1_^−^] to ^3^[P_680_^+^Pheo_D1_^−^], the excess excitation energy dissipated through the non-radiative triplet route (Fig. 9). Experiments suggest that the observable triplet in the closed RC has an extremely short lifetime (*t*_1/2_ < 20 μs), and it has been proposed that this is because Q_A_^−^ quenches RC triplet states through ^3^Pheo_D1_.^32^ However, this mechanism of triplet quenching involving the semiquinone (Q_A_^−^) and ^3^Pheo_D1_ is not well understood. Based on our computed excitation profile of the closed RC (Fig. 9), we find numerous thermodynamically accessible triplet states that are localized on the individual pigments (Chl_D1_, P_D1_, P_D2_ and Pheo_D1_). All these local excitations are in fact lower in energy than the CT ^3^[Chl_D1_^*δ*+^Pheo_D1_^*δ*−^] and ^3^[P_D1_^*δ*+^Pheo_D1_^*δ*+^] excitations, which is in contrast to the triplet energy profile of open RC [S_1_Q_A_] (see Fig. 8). Specifically, all D1 pigments in the closed RC possess at least two triplet excitations (T_1_ to T_8_) energetically lower than the first CT state. Thus, non-radiative energy dissipation involving multiple RC pigments might be a possibility in the closed RC, in line with arguments regarding triplet delocalization pathways discussed in recent FTIR studies.^87^ When Q_A_ is doubly reduced as Q_A_H_2_, the Pheo_D1_^−^ anion is expected to be more stable in the absence of a negative charge in its vicinity.^25,45,46,130^ This can stabilize ^1^[P_680_^+^Pheo_D1_^−^] and a subsequent spin inversion to ^3^[P_680_^+^Pheo_D1_^−^] may again lead to more centers favoring the triplet route as opposed to a direct charge recombination to the singlet state.

It is known that formation of triplet states is detrimental to photosynthetic organisms as long-lived triplets in the RC can accelerate the formation of reactive oxygen species and subsequent photodamage.^43,49,51^ In this respect, we provided a quantitative explanation of how the PSII protein matrix and redox active cofactors may work in tandem to tune the energetics of primary charge separation and triplet formation in photosynthetic reaction centers. Our results have implications for photoprotection mechanisms in both the open and the closed states of active PSII. The next line of photoprotection in the RC may involve the delocalization of triplet states away from Chl_D1_ onto other pigments at ambient temperatures to avoid the selective damage of the D1 protein. However, if this still leads to photoinactivation, the D1 protein is selectively degraded and regenerated, thereby allowing photosynthetic organisms to preserve functionality even under extreme conditions.^48,131,132^

## 4. Conclusions

This work provides a detailed overview of the low-energy excitation spectrum of the PSII-RC, explaining the asymmetry of singlet-triplet excitations and charge transfer states along the D1 and D2 branches. The PSII protein environment explicitly controls the excitonic asymmetry of the RC, leading to low-energy charge-transfer excitations and triplet formation on the D1 side. Based on our calculations we may speculate that the observable triplet state at the RC is unlikely to be formed by usual inter-system crossing (ISC), but through a radical-pair charge recombination preceding ISC. However, this cannot yet be concluded with confidence given that the kinetics of triplet formation and the factors/processes that determine them are not addressed in the present work. We identified all low-lying charge transfer excitations in the pigment assemblies, and find that the lowest triplet exciton is localized on Chl_D1_. With supporting insights from EPR calculations we confirm the localization of the most stable triplet state on Chl_D1_. Furthermore, we present the first theoretical description of the excited state properties of the “closed RC” (S_2_Q_A_^−^), explicitly quantifying the electrostatic effect of semiquinone (Q_A_^−^) and how it influences excited state properties of RC pigments. Overall, this work provides a refined basis for the electronic-level understanding of primary and secondary electron transfer pathways, offering detailed electronic structure information as a foundation for discussing possible photoprotection mechanisms in oxygenic photosynthesis.

## Author contributions

S. B.: methodology, investigation, analysis, writing – original draft; F. N.: resources, supervision; D. A. P.: conceptualization, methodology, supervision, writing – review and editing.

## Conflicts of interest

There are no conflicts to declare.

## Supplementary Material

SC-014-D3SC02985A-s001
