# Supplementary material for: Triplet states in the reaction center of Photosystem II
Source: Chem Sci. 2023 Aug 17;14(35):9503–16. doi: 10.1039/d3sc02985a (PMC10498673; doi:10.1039/d3sc02985a)
Supplement: SC-014-D3SC02985A-s001 [file SC-014-D3SC02985A-s001.pdf]

Electronic Supplementary Material (ESI) for Chemical Science.
This journal is © The Royal Society of Chemistry 2023

Electronic Supplementary Information

Triplet States in the Reaction Center of Photosystem II

Sinjini Bhattacharjee, Frank Neese and Dimitrios A. Pantazis

Max-Planck-Institut für Kohlenforschung, Kaiser-Wilhelm-Platz 1, 45470 Mülheim an der Ruhr, Germany.

Contents:

Tables: S1 to S9

Figures: S1 to S4

1. EXCITATION ENERGIES

The singlet excitation energies are computed using full TDDFT without employing the Tamm–Dancoff approximation (TDA). Vertical triplet excitation energies were computed on the pair-optimized ground state singlet geometries, using the TDA approximation on TD-DFT.

Table S1. Excitation energies (ωB97X-D3BJ/def2-TZVP): **Chl$_{D1}$ (gas-phase)**

Roots	E_S (eV)	f_{osc}	Transition	E_T (eV)	Transition
1	1.884	0.24	HOMO → LUMO (0.81)<br>HOMO-1→LUMO+1 (0.13)	1.290	HOMO → LUMO (0.90)<br>HOMO-1→ LUMO+1 (0.02)
2	2.387	0.04	HOMO-1 → LUMO (0.69)<br>HOMO → LUMO+1 (0.26)	1.763	HOMO-1 → LUMO (0.89)
3	3.382	0.73	HOMO → LUMO+1 (0.59)<br>HOMO-1 → LUMO (0.19)	2.212	HOMO → LUMO+1 (0.88)

S1-T1 Gap: 0.594 eV

Table S2. Excitation energies (ωB97X-D3BJ/def2-TZVP) and QM/MM: **Chl$_{D1}$ (in protein)**

Roots	E_S (eV)	f_{osc}	Transition	E_T (eV)	Transition
1	1.818	0.29	HOMO → LUMO (0.84)<br>HOMO-1→ LUMO+1 (0.11)	1.220	HOMO → LUMO (0.92)
2	2.376	0.05	HOMO-1 → LUMO (0.72)<br>HOMO → LUMO+1 (0.23)	1.727	HOMO-1 → LUMO (0.90)
3	3.425	0.82	HOMO → LUMO+1 (0.70)<br>HOMO-1 → LUMO (0.21)	2.280	HOMO → LUMO+1 (0.89)

S1-T1 Gap: 0.598 eV

Table S3. Excitation energies (ωB97X-D3BJ/def2-TZVP) and QM/MM: **Chl$_{D1}$ + M172 (in protein)**

Roots	E_S (eV)	f_{osc}	Transition	E_T (eV)	Transition
1	1.819	0.28	HOMO → LUMO (0.84)<br>HOMO-1→ LUMO+1 (0.11)	1.222	HOMO → LUMO (0.92)
2	2.376	0.05	HOMO-1 → LUMO (0.72)<br>HOMO → LUMO+1 (0.22)	1.727	HOMO-1 → LUMO (0.89)
3	3.410	0.85	HOMO → LUMO+1 (0.70)<br>HOMO-1 → LUMO (0.20)	2.282	HOMO → LUMO+1 (0.89)

S1-T1 Gap: 0.597 eV

Table S4. Excitation energies (ωB97X-D3BJ/def2-TZVP): **Chl$_{D2}$ (gas-phase)**

Roots	E_S (eV)	f_{osc}	Transition	E_T (eV)	Transition
1	1.900	0.24	HOMO → LUMO (0.79)<br>HOMO-1→ LUMO+1 (0.13)	1.319	HOMO → LUMO (0.88)<br>HOMO-1→ LUMO+1 (0.03)
2	2.395	0.04	HOMO-1 → LUMO (0.66)<br>HOMO → LUMO+1 (0.25)	1.745	HOMO-1 → LUMO (0.88)
3	3.388	0.73	HOMO → LUMO+1 (0.62)<br>HOMO-1 → LUMO (0.20)	2.224	HOMO → LUMO+1 (0.87)

S1-T1 Gap: 0.581 eV

Table S5. Excitation energies (ωB97X-D3BJ/def2-TZVP) and QM/MM: **Chl_{D2} (in protein)**

Root	E_S (eV)	f_{osc}	Transition	E_T (eV)	Transition
1	1.878	0.25	HOMO → LUMO (0.81)<br>HOMO-1→ LUMO+1 (0.12)	1.288	HOMO → LUMO (0.89)
2	2.406	0.05	HOMO-1 → LUMO (0.68)<br>HOMO → LUMO+1 (0.24)	1.760	HOMO-1 → LUMO (0.88)
3	3.419	0.80	HOMO → LUMO+1 (0.65)<br>HOMO-1 → LUMO (0.21)	2.250	HOMO → LUMO+1 (0.88)

S1-T1 Gap: 0.59 eV

Table S6. Singlet and triplet excitation energies (ωB97X-D3BJ/def2-TZVP): **P_{D1}–P_{D2}** pair **(in protein)**. LE indicates local excitations. Arrows indicate charge transfer (CT) excitations.

Roots	E_S (eV)	f_{osc}	Transition	E_T (eV)	Transition
1	1.859	0.41	LE (P_{D1}) + LE (P_{D2})	1.291	LE (P_{D2})
2	1.885	0.07	LE (P_{D1}) + LE (P_{D2})	1.305	LE (P_{D1})
3	2.416	0.02	LE (P_{D1}) + LE (P_{D2})	1.773	LE (P_{D1})
4	2.427	0.08	LE (P_{D1}) + LE (P_{D2})	1.781	LE (P_{D2})
5	3.010	0.06	CT (P_{D1} → P_{D2})	2.270	LE (P_{D2})
6	3.188	0.33	CT (P_{D2} → P_{D1}) + LE (P_{D2})	2.278	LE (P_{D1})

Table S7. Singlet and triplet excitation energies (ωB97X-D3BJ/def2-TZVP): **P_{D1}–P_{D2}–Chl_{D2}–$Pheo_{D2}$** tetramer **(open RC, S_1Q_A).**

Roots	E_S (eV)	f_{osc}	Transition	E_T (eV)	Transition
1	1.706	0.00	CT (P_{D1} → $Pheo_{D2}$)	1.279	LE (Chl_{D2})
2	1.816	0.00	CT (P_{D2} → $Pheo_{D2}$)	1.287	LE (P_{D2})
3	1.841	0.63	LE (Chl_{D2}) + LE (P_{D1})	1.300	LE (P_{D1})
4	1.854	0.10	LE (Chl_{D2}) + LE (P_{D2})	1.390	LE ($Pheo_{D2}$)
5	1.885	0.06	LE (P_{D1}) + LE (P_{D2})	1.674	LE ($Pheo_{D2}$)
6	2.032	0.01	CT (Chl_{D2} → $Pheo_{D2}$)	1.706	CT (P_{D1} → $Pheo_{D2}$)
7	2.047	0.16	LE ($Pheo_{D2}$)	1.759	LE (P_{D1})
8	2.219	0.00	P_{D1} → $Pheo_{D2}$ + Chl_{D2} → $Pheo_{D2}$	1.763	LE (Chl_{D2})
9	2.297	0.00	CT (P_{D2} →$Pheo_{D2}$)	1.779	LE (P_{D2})
10	2.407	0.01	LE (Chl_{D2})	1.816	CT (P_{D2} → $Pheo_{D2}$)
11	2.411	0.03	LE (P_{D2})	2.032	CT (Chl_{D2} → $Pheo_{D2}$)
12	2.418	0.14	LE (P_{D2})	2.219	CT (P_{D1} → $Pheo_{D2}$)

Table S8. Singlet and triplet excitation energies (ωB97X-D3BJ/def2-TZVP): **P_{D1}–P_{D2}–Chl_{D1}–$Pheo_{D1}$** tetramer **(closed RC, $S_2Q_A^-$)**

Roots	E_S (eV)	f_{osc}	Transition	E_T (eV)	Transition
1	1.796	0.40	LE (Chl_{D1})	1.223	LE (Chl_{D1})
2	1.862	0.37	LE (P_{D1}) + LE (P_{D2})	1.296	LE (P_{D2})
3	1.888	0.05	LE (P_{D1}) + LE (P_{D2})	1.306	LE (P_{D1})
4	1.990	0.18	LE ($Pheo_{D1}$)	1.394	LE ($Pheo_{D1}$)
5	2.231	0.00	CT (Chl_{D1} → $Pheo_{D1}$)	1.670	LE ($Pheo_{D1}$)
6	2.276	0.00	CT (P_{D1} → $Pheo_{D1}$)	1.729	LE (Chl_{D1})
7	2.376	0.04	LE (Chl_{D1})	1.769	LE (P_{D1})

8	2.405	0.02	LE (P_{D1}) + LE (P_{D2})	1.770	LE (P_{D2})
9	2.421	0.21	LE (P_{D1}) + LE (P_{D2})	2.227	CT (Chl_{D1} →$Pheo_{D1}$)
10	2.425	0.03	LE ($Pheo_{D1}$)	2.256	LE (P_{D2})
11	2.436	0.00	CT (P_{D2} → $Pheo_{D1}$) (0.99)	2.276	CT (P_{D1} → $Pheo_{D1}$)
12	2.732	0.00	CT (P_{D1} → $Pheo_{D1}$) (0.98)	2.277	CT (P_{D1} → $Pheo_{D2}$) + LE (P_{D1})

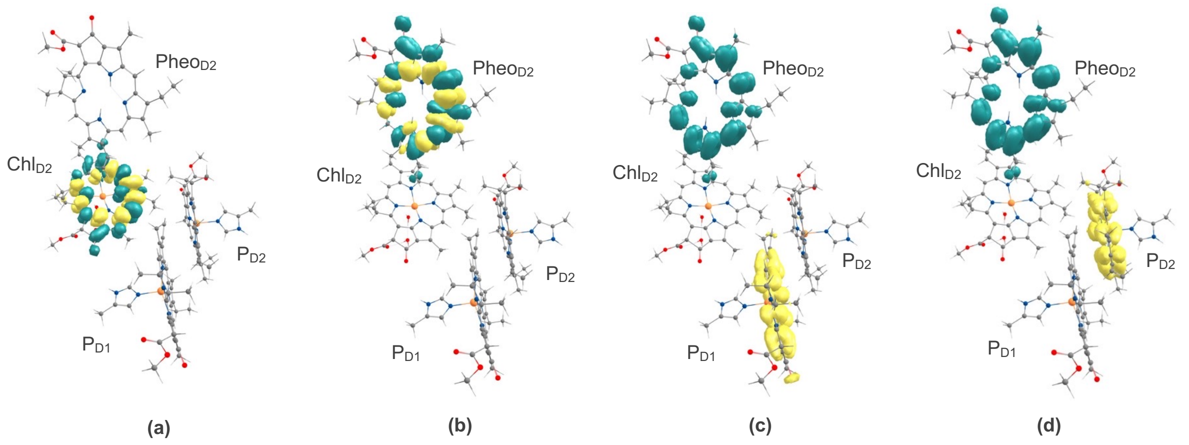

Figure S1. Difference densities describing the lowest singlet-triplet excitations of the D2 branch in PSII: (a) local $^3Chl_{D2}$ excitation; (b) local $^3Pheo_{D2}$ excitation; (c) the lowest triplet excitation with $^3[P_{D1}{}^{\delta+}Pheo_{D2}{}^{\delta-}]$ charge transfer character; (d) the lowest triplet excitation with $^3[P_{D2}{}^{\delta+}Pheo_{D2}{}^{\delta-}]$ charge transfer character.

2. EPR PROPERTIES

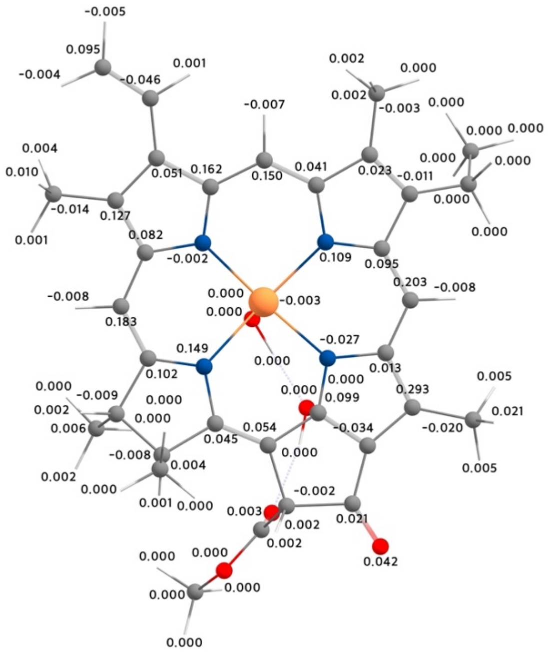

Figure S2. Mulliken spin population analysis of Chl_{D1} in protein calculated with DFT-TPSSh and QM/MM with EPR-II basis set on H-atoms and def2-TZVP on the remaining atoms.

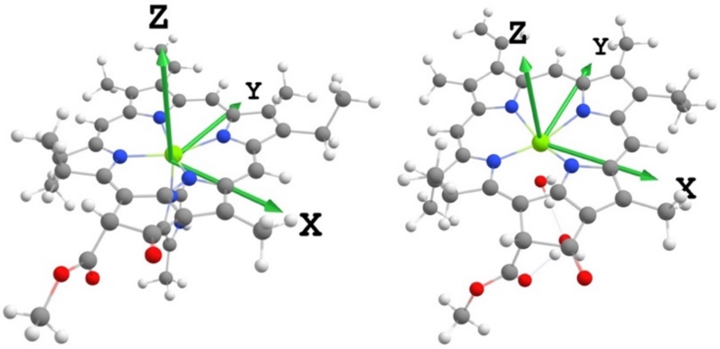

Figure S3. The orientation of the ZFS D-tensor in P_{D1} and Chl_{D1} RC pigments, computed with DFT-TPSSh and QM/MM with EPR-II basis set on H-atoms and def2-TZVP on the remaining atoms.

Table S9. Computed and experimental ZFS parameters *D* and *E* and principal values of the *g*-tensors for RC pigments in their triplet states. All values are calculated using gauge including atomic orbitals (GIAOs).

**EPR/ENDOR**		***D*** (cm^{-1})	***E/D***	g_{xx}	g_{yy}	g_{zz}	g_{iso}
^{3}P680	Niklas *et al.*, 2022[34]	0.0288	0.15	2.00310	2.00320	2.00220	2.00280
	Pashenko *et al.*, 2003[58]	0.0289	0.15	2.00324	2.00306	2.00231	2.00287
**QM/MM**		***D*** (cm^{-1})	***E/D***	***g*-shifts** (ppm)			
**Pigment**	**Method**			Δg_{xx}	Δg_{yy}	Δg_{zz}	g_{iso}
Chl_{D1}	TPSSh	0.0189	0.31	1077	1774	-383	822
	B3LYP (10% HF)			572	1331	-378	508
Chl_{D2}	TPSSh	0.0194	0.28	1054	1753	-446	787
	B3LYP (10% HF)			527	1292	-427	464
P_{D1}	TPSSh	0.0198	0.31	448	1250	-228	490
	B3LYP (10% HF)			524	1386	-288	541
P_{D2}	TPSSh	0.0199	0.28	990	1827	-476	780
	B3LYP (10% HF)			483	1425	-621	429

The calculated *g*-values g_{ii} ($i = x, y, z$) are given as *g*-shifts Δg_{ii} in parts per million (ppm) with $\Delta g_{ii} = 10^6 \times (g_i - g_e)$, where $g_e = 2.002319$ is the free electron *g*-value. The isotropic *g*-value is defined as one-third of the sum of the principal *g*-values.

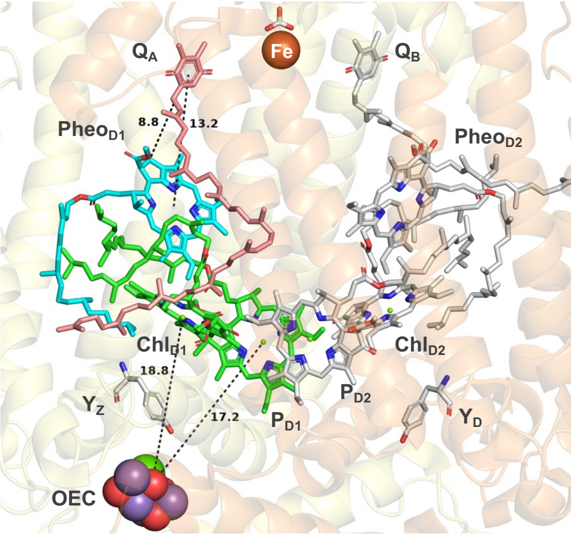

Figure S4. Depiction of distances (in Å) of the redox active cofactors QA and OEC from the P_{D1}, Chl_{D1}, and $Pheo_{D1}$ pigments in the PSII reaction center. Distances are measured from the center of each chlorin ring, unless otherwise specified.

QM/MM optimized geometries (PBE/def2-TZVP) of RC pigments in their triplet (*S*=1) states.

Chl$_{D1}$ (79)

C	-10.714701	-6.0341182	-7.576730
C	-4.612783	-12.431747	-6.165519
C	-2.123136	-6.310311	-1.770266
C	-9.426704	-3.106103	-3.547280
N	-8.892446	-7.577800	-5.280435
C	-4.632982	-13.591884	-6.862608
C	-1.802921	-7.219797	-0.572554
C	-10.319420	-4.094031	-4.403490
N	-6.662156	-9.514096	-5.240856
O	-9.857050	-2.016454	-3.169031
C	-11.489680	-4.542925	-3.557698
N	-7.100446	-5.743906	-3.804566
C	-13.825346	-4.947867	-3.518084
C	-9.436812	-5.306362	-4.656321
C	-8.931884	-9.867377	-6.142594
C	-4.259933	-9.716394	-4.735050
C	-4.971538	-5.366666	-2.692356
C	-11.932039	-8.868262	-5.242127
C	-7.724471	-12.598749	-7.081157
C	-1.486327	-8.896169	-3.580879
C	-6.319466	-2.509665	-1.992069
C	-9.734486	-6.469288	-5.303022
C	-7.611546	-10.289034	-5.894480
C	-4.035149	-8.531834	-4.067170
C	-6.248909	-4.925818	-3.051500
O	-11.387920	-4.986996	-2.425624
C	-10.916213	-6.729150	-6.213750
C	-7.023627	-11.522327	-6.330721
C	-2.766277	-8.147302	-3.446235
C	-6.892398	-3.660530	-2.742796
O	-12.663519	-4.436737	-4.213706
C	-10.944257	-8.272196	-6.257562
C	-5.669990	-11.475472	-5.959718
C	-3.012854	-6.990119	-2.756887
C	-8.171549	-3.766783	-3.334452
C	-9.505229	-8.614917	-5.885658
C	-5.492154	-10.199942	-5.279361
C	-4.392539	-6.600562	-3.070386
C	-8.215993	-5.043223	-3.959329
N	-4.999328	-7.563320	-3.823624
Mg	-7.060797	-7.785258	-4.131092
H	-10.680849	-4.945137	-7.432317
H	-11.552956	-6.251331	-8.253923
H	-3.663746	-12.180896	-5.681106
H	-2.595511	-5.389086	-1.412214
H	-1.203736	-5.980940	-2.265350
H	-5.514350	-13.955551	-7.384874
H	-3.741958	-14.217930	-6.908425
H	-1.054151	-6.759790	0.083036
H	-1.404680	-8.188612	-0.893319
H	-2.706939	-7.414979	0.016521
H	-10.681675	-3.578541	-5.305072
H	-14.675127	-4.356430	-3.866375
H	-13.702881	-4.844995	-2.436043

H	-13.951814	-6.005357	-3.786240
H	-9.582732	-10.592376	-6.632113
H	-3.401603	-10.383651	-4.811998
H	-4.366416	-4.703380	-2.076339
H	-11.877591	-9.965667	-5.231995
H	-12.964391	-8.573819	-5.484556
H	-11.699818	-8.515716	-4.226625
H	-8.815873	-12.492106	-7.030993
H	-7.459594	-13.588214	-6.685565
H	-7.439035	-12.604366	-8.146458
H	-1.613507	-9.963703	-3.352818
H	-0.711702	-8.506269	-2.912861
H	-1.095787	-8.820205	-4.607728
H	-5.813075	-1.802925	-2.670395
H	-5.580940	-2.836618	-1.250232
H	-7.102946	-1.939660	-1.475181
H	-11.848710	-6.328664	-5.788766
H	-11.199696	-8.641485	-7.261965
O	-8.098416	-8.387873	-2.440735
H	-8.583697	-7.648752	-1.959119
H	-7.670561	-8.891640	-1.712385
O	-9.284650	-6.399856	-1.097387
H	-9.964868	-5.809392	-1.489233
H	-9.334003	-6.282539	-0.127030
H	-9.767057	-6.300462	-8.109780

Chl_{D2} (79)

C	6.377000	7.996000	-8.738000
C	0.670000	15.154000	-7.088000
C	-2.337000	9.107000	-2.931000
C	4.962000	5.615000	-4.381000
N	4.487000	9.848000	-6.629000
C	0.670000	16.277000	-7.837000
C	-2.459000	9.771000	-1.550000
C	5.861000	6.477000	-5.370000
N	2.430000	11.981000	-6.463000
O	5.384000	4.590000	-3.849000
C	7.058000	6.994000	-4.600000
N	2.672000	8.248000	-4.924000
C	9.414000	7.038000	-4.403000
C	4.988000	7.655000	-5.762000
C	4.599000	12.069000	-7.646000
C	0.161000	12.491000	-5.645000
C	0.543000	8.034000	-3.748000
C	7.575000	11.106000	-6.853000
C	3.603000	14.841000	-8.565000
C	-2.617000	11.942000	-4.320000
C	1.816000	5.256000	-2.783000
C	5.305000	8.724000	-6.546000
C	3.358000	12.619000	-7.276000
C	-0.157000	11.317000	-4.997000
C	1.812000	7.537000	-4.076000
O	6.982000	7.703000	-3.607000
C	6.505000	8.863000	-7.466000
C	2.864000	13.905000	-7.676000
C	-1.439000	11.029000	-4.354000
C	2.435000	6.303000	-3.639000

O	8.233000	6.606000	-5.130000
C	6.532000	10.388000	-7.723000
C	1.602000	14.051000	-7.084000
C	-1.323000	9.784000	-3.795000
C	3.711000	6.312000	-4.253000
C	5.119000	10.805000	-7.334000
C	1.367000	12.818000	-6.348000
C	0.024000	9.295000	-4.131000
C	3.772000	7.505000	-5.021000
N	0.702000	10.234000	-4.848000
Mg	2.745000	10.252000	-5.362000
H	6.451000	6.930000	-8.483000
H	7.194000	8.228000	-9.437000
H	-0.141000	15.068000	-6.358000
H	-2.096000	8.042000	-2.814000
H	-3.314000	9.137000	-3.436000
H	1.419000	16.482000	-8.598000
H	-0.097000	17.033000	-7.684000
H	-2.722000	10.833000	-1.642000
H	-1.517000	9.707000	-0.993000
H	-3.236000	9.286000	-0.949000
H	6.196000	5.836000	-6.199000
H	9.317000	6.803000	-3.338000
H	9.555000	8.120000	-4.525000
H	10.245000	6.471000	-4.834000
H	5.250000	12.707000	-8.245000
H	-0.617000	13.254000	-5.649000
H	-0.093000	7.410000	-3.121000
H	7.495000	12.197000	-6.949000
H	8.593000	10.806000	-7.143000
H	7.432000	10.851000	-5.793000
H	3.334000	14.680000	-9.620000
H	4.689000	14.694000	-8.492000
H	3.387000	15.887000	-8.323000
H	-2.428000	12.822000	-3.684000
H	-3.504000	11.437000	-3.915000
H	-2.876000	12.304000	-5.327000
H	1.557000	4.360000	-3.368000
H	0.885000	5.611000	-2.327000
H	2.498000	4.919000	-1.989000
H	7.428000	8.541000	-6.959000
H	6.731000	10.620000	-8.781000
O	3.877000	10.825000	-3.740000
H	4.058000	11.723000	-3.424000
H	4.296000	10.167000	-3.079000
O	4.908000	9.025000	-2.187000
H	5.068000	9.058000	-1.220000
H	5.625000	8.487000	-2.592000
H	5.411286	8.136000	-9.283714

P_{D1}-P_{D2} (170)

C	6.553000	-1.286000	-2.259000
H	6.675000	-2.320000	-1.913000
H	7.187000	-0.631000	-1.648000
C	5.119000	-0.880000	-2.160000
N	4.722000	0.418000	-1.905000
H	5.314000	1.240000	-1.735000

C	3.373000	0.468000	-1.892000
H	2.806000	1.376000	-1.714000
N	2.860000	-0.738000	-2.116000
C	3.939000	-1.586000	-2.287000
H	3.805000	-2.648000	-2.471000
C	-11.010000	3.480000	-1.950000
H	-11.270000	4.417000	-1.446000
H	-11.450000	2.661000	-1.366000
C	-9.527000	3.313000	-2.053000
N	-8.891000	2.176000	-1.604000
H	-9.349000	1.336000	-1.230000
C	-7.569000	2.286000	-1.852000
H	-6.840000	1.526000	-1.588000
N	-7.301000	3.448000	-2.444000
C	-8.515000	4.095000	-2.578000
H	-8.581000	5.085000	-3.022000
C	-0.775000	-4.491000	-5.718000
C	-1.544000	3.870000	-2.725000
C	-0.178000	-0.595000	3.730000
C	1.395000	-6.493000	-1.738000
N	0.599000	-2.216000	-3.850000
C	-2.152000	4.783000	-3.517000
C	-1.592000	-0.579000	4.330000
C	1.379000	-5.946000	-3.267000
N	-0.183000	0.445000	-2.786000
O	1.392000	-7.681000	-1.456000
C	2.628000	-6.437000	-3.930000
N	0.935000	-3.051000	-1.046000
C	3.500000	-8.430000	-4.885000
C	1.170000	-4.457000	-3.143000
C	0.052000	-0.215000	-5.152000
C	-0.716000	1.661000	-0.712000
C	0.688000	-2.523000	1.332000
C	2.090000	-2.142000	-6.793000
C	-0.915000	2.714000	-5.679000
C	-0.990000	2.137000	2.360000
C	1.405000	-5.637000	1.660000
C	0.855000	-3.555000	-4.117000
C	-0.265000	0.716000	-4.148000
C	-0.358000	0.724000	0.239000
C	0.966000	-3.433000	0.301000
O	3.707000	-5.870000	-4.005000
C	0.622000	-3.859000	-5.584000
C	-0.754000	2.046000	-4.360000
C	-0.513000	0.897000	1.683000
C	1.234000	-4.857000	0.412000
O	2.387000	-7.689000	-4.381000
C	0.707000	-2.450000	-6.210000
C	-1.006000	2.586000	-3.092000
C	-0.136000	-0.285000	2.267000
C	1.332000	-5.309000	-0.918000
C	0.420000	-1.556000	-5.011000
C	-0.625000	1.553000	-2.137000
C	0.276000	-1.178000	1.170000
C	1.162000	-4.164000	-1.741000
N	0.149000	-0.539000	-0.033000
Mg	0.786000	-1.202000	-1.931000

H	-1.543000	-3.746000	-5.460000
H	-0.894000	-5.342000	-5.034000
H	-1.482000	4.110000	-1.661000
H	0.437000	0.152000	4.260000
H	0.286000	-1.568000	3.932000
H	-2.336000	4.609000	-4.575000
H	-2.551000	5.702000	-3.090000
H	-2.227000	-1.366000	3.904000
H	-2.099000	0.380000	4.154000
H	-1.546000	-0.729000	5.414000
H	0.567000	-6.466000	-3.793000
H	3.601000	-8.254000	-5.965000
H	3.264000	-9.483000	-4.685000
H	4.423000	-8.140000	-4.371000
H	-0.052000	0.139000	-6.179000
H	-1.155000	2.581000	-0.329000
H	0.740000	-2.894000	2.357000
H	2.319000	-2.807000	-7.640000
H	2.866000	-2.270000	-6.025000
H	2.125000	-1.104000	-7.147000
H	-0.453000	2.137000	-6.491000
H	-0.444000	3.707000	-5.664000
H	-1.977000	2.867000	-5.936000
H	-0.861000	2.074000	3.446000
H	-2.055000	2.328000	2.156000
H	-0.437000	3.021000	2.012000
H	0.671000	-6.454000	1.742000
H	1.316000	-4.987000	2.537000
H	2.393000	-6.120000	1.695000
H	1.399000	-4.531000	-5.973000
H	-0.054000	-2.293000	-6.992000
C	-4.407000	7.528000	-6.667000
C	-3.203000	-1.106000	-3.792000
C	-4.737000	3.020000	2.866000
C	-5.954000	9.261000	-2.292000
N	-5.329000	5.072000	-4.629000
C	-3.556000	-2.266000	-4.370000
C	-6.117000	3.622000	3.206000
C	-6.096000	8.763000	-3.819000
N	-4.514000	2.352000	-3.709000
O	-6.056000	10.426000	-1.951000
C	-7.510000	9.008000	-4.287000
N	-5.232000	5.829000	-1.774000
C	-8.954000	10.583000	-5.281000
C	-5.740000	7.289000	-3.772000
C	-4.856000	3.109000	-6.023000
C	-4.005000	1.002000	-1.711000
C	-4.884000	5.198000	0.563000
C	-7.162000	4.968000	-7.386000
C	-3.670000	0.265000	-6.693000
C	-3.988000	0.280000	1.335000
C	-5.322000	8.336000	1.035000
C	-5.621000	6.397000	-4.819000
C	-4.463000	2.150000	-5.055000
C	-4.254000	1.926000	-0.678000
C	-5.132000	6.165000	-0.427000
O	-8.445000	8.251000	-4.110000

C	-5.676000	6.745000	-6.298000
C	-3.971000	0.810000	-5.336000
C	-4.251000	1.625000	0.748000
C	-5.377000	7.587000	-0.243000
O	-7.616000	10.208000	-4.901000
C	-5.743000	5.342000	-6.945000
C	-3.737000	0.211000	-4.105000
C	-4.524000	2.813000	1.398000
C	-5.655000	8.068000	-1.527000
C	-5.264000	4.426000	-5.821000
C	-4.073000	1.205000	-3.094000
C	-4.670000	3.826000	0.354000
C	-5.540000	6.961000	-2.414000
N	-4.536000	3.245000	-0.887000
Mg	-5.232000	4.015000	-2.720000
H	-3.528000	6.892000	-6.505000
H	-4.289000	8.417000	-6.034000
H	-2.457000	-1.131000	-2.990000
H	-3.952000	3.664000	3.292000
H	-4.630000	2.050000	3.373000
H	-4.338000	-2.305000	-5.128000
H	-3.122000	-3.214000	-4.051000
H	-6.866000	3.326000	2.456000
H	-6.071000	4.718000	3.248000
H	-6.477000	3.277000	4.180000
H	-5.422000	9.388000	-4.422000
H	-9.240000	10.047000	-6.194000
H	-8.935000	11.664000	-5.446000
H	-9.669000	10.327000	-4.492000
H	-4.842000	2.771000	-7.063000
H	-3.710000	0.003000	-1.391000
H	-4.863000	5.541000	1.598000
H	-7.540000	5.673000	-8.143000
H	-7.843000	4.998000	-6.522000
H	-7.176000	3.953000	-7.805000
H	-3.129000	-0.686000	-6.604000
H	-3.041000	0.964000	-7.269000
H	-4.579000	0.085000	-7.292000
H	-4.603000	-0.504000	0.868000
H	-4.188000	0.260000	2.414000
H	-2.933000	-0.006000	1.200000
H	-5.954000	9.231000	1.016000
H	-4.300000	8.671000	1.273000
H	-5.639000	7.694000	1.861000
H	-6.576000	7.338000	-6.529000
H	-5.061000	5.273000	-7.803000
H	6.949429	-1.301000	-3.296143
H	-11.489286	3.517857	-2.952143
H	-1.029286	-4.855286	-6.733000
H	-4.385571	7.881571	-7.721286

Pheo$_{D1}$ (74)

C	-5.852000	-14.232000	-15.319000
C	-4.940000	-7.464000	-9.082000
C	0.197000	-5.407000	-14.763000
C	-2.831000	-12.010000	-18.509000
N	-5.345000	-10.955000	-14.540000

C	-5.594000	-7.514000	-7.894000
C	1.492000	-6.237000	-14.734000
C	-4.062000	-12.650000	-17.726000
N	-4.934000	-8.969000	-12.393000
O	-2.321000	-12.515000	-19.509000
C	-5.222000	-12.888000	-18.658000
N	-2.885000	-9.690000	-15.884000
C	-7.020000	-11.906000	-19.834000
C	-4.301000	-11.720000	-16.551000
C	-6.633000	-10.786000	-12.480000
C	-3.541000	-7.025000	-11.800000
C	-1.251000	-7.884000	-15.987000
C	-8.645000	-11.736000	-14.848000
C	-7.075000	-9.851000	-9.510000
C	-1.429000	-4.804000	-12.022000
C	-0.444000	-9.438000	-18.689000
C	-5.315000	-11.794000	-15.580000
C	-5.921000	-9.738000	-11.812000
C	-2.704000	-6.909000	-12.925000
C	-1.834000	-9.057000	-16.538000
O	-5.472000	-13.963000	-19.183000
C	-6.428000	-12.834000	-15.565000
C	-6.134000	-9.268000	-10.499000
C	-1.658000	-5.875000	-13.030000
C	-1.526000	-9.776000	-17.724000
O	-5.933000	-11.769000	-18.889000
C	-7.309000	-12.333000	-14.394000
C	-5.243000	-8.176000	-10.290000
C	-0.992000	-6.117000	-14.199000
C	-2.459000	-10.841000	-17.765000
C	-6.413000	-11.296000	-13.730000
C	-4.510000	-7.989000	-11.528000
C	-1.646000	-7.283000	-14.802000
C	-3.296000	-10.743000	-16.619000
N	-2.692000	-7.736000	-13.999000
H	-4.614000	-9.083000	-13.352000
H	-3.231000	-9.301000	-14.993000
H	-5.455000	-14.263000	-14.297000
H	-5.027000	-14.471000	-16.002000
H	-4.032000	-6.856000	-9.125000
H	0.361000	-4.479000	-14.194000
H	-0.015000	-5.094000	-15.797000
H	-6.502000	-8.091000	-7.735000
H	-5.213000	-6.969000	-7.030000
H	2.352000	-5.624000	-15.027000
H	1.441000	-7.104000	-15.406000
H	1.686000	-6.616000	-13.722000
H	-3.722000	-13.646000	-17.403000
H	-7.838000	-12.479000	-19.384000
H	-6.673000	-12.405000	-20.747000
H	-7.343000	-10.884000	-20.054000
H	-7.455000	-11.220000	-11.913000
H	-3.368000	-6.298000	-11.007000
H	-0.414000	-7.448000	-16.529000
H	-8.486000	-10.897000	-15.542000
H	-9.215000	-11.364000	-13.986000
H	-9.258000	-12.495000	-15.353000

H	-7.845000	-9.130000	-9.201000
H	-6.531000	-10.143000	-8.602000
H	-7.583000	-10.740000	-9.903000
H	-0.609000	-4.142000	-12.324000
H	-1.175000	-5.217000	-11.034000
H	-2.334000	-4.189000	-11.900000
H	0.477000	-9.157000	-18.158000
H	-0.714000	-8.582000	-19.327000
H	-0.232000	-10.293000	-19.344000
H	-6.982000	-12.807000	-16.518000
H	-7.513000	-13.150000	-13.688000
H	-6.603235	-15.038515	-15.445151

Pheo$_{D2}$ (74)

C	1.250000	15.650000	-17.092000
C	0.712000	9.491000	-10.323000
C	-4.963000	7.320000	-15.504000
C	-1.454000	12.886000	-20.296000
N	0.808000	12.549000	-16.052000
C	1.369000	9.738000	-9.163000
C	-6.357000	7.947000	-15.667000
C	-0.148000	13.503000	-19.625000
N	0.332000	10.952000	-13.645000
O	-1.800000	13.158000	-21.447000
C	1.059000	13.153000	-20.473000
N	-1.855000	11.373000	-17.154000
C	2.671000	11.480000	-20.933000
C	-0.145000	12.958000	-18.213000
C	2.219000	12.511000	-14.070000
C	-1.210000	9.245000	-12.747000
C	-3.673000	9.779000	-16.906000
C	4.058000	13.049000	-16.682000
C	2.960000	11.666000	-11.138000
C	-3.249000	6.937000	-12.796000
C	-4.237000	10.756000	-19.959000
C	0.809000	13.208000	-17.218000
C	1.477000	11.603000	-13.249000
C	-2.153000	9.107000	-13.785000
C	-2.975000	10.746000	-17.686000
O	1.531000	13.853000	-21.350000
C	1.893000	14.267000	-17.314000
C	1.799000	11.186000	-11.940000
C	-3.165000	8.035000	-13.798000
C	-3.147000	11.173000	-19.030000
O	1.535000	11.915000	-20.167000
C	2.837000	13.826000	-16.171000
C	0.823000	10.224000	-11.554000
C	-3.894000	8.204000	-14.945000
C	-2.048000	12.040000	-19.294000
C	1.927000	12.932000	-15.340000
C	-0.100000	10.088000	-12.663000
C	-3.301000	9.349000	-15.638000
C	-1.270000	12.123000	-18.109000
N	-2.251000	9.884000	-14.895000
H	-0.091000	11.065000	-14.564000
H	-1.568000	11.157000	-16.185000
H	0.897000	15.726000	-16.056000

H	0.384000	15.812000	-17.748000
H	-0.016000	8.675000	-10.315000
H	-5.044000	6.431000	-14.862000
H	-4.624000	6.950000	-16.484000
H	2.074000	10.554000	-9.031000
H	1.164000	9.131000	-8.282000
H	-6.983000	7.312000	-16.309000
H	-6.311000	8.943000	-16.129000
H	-6.878000	8.043000	-14.707000
H	-0.238000	14.597000	-19.656000
H	2.405000	11.356000	-21.993000
H	2.959000	10.516000	-20.500000
H	3.496000	12.200000	-20.850000
H	3.130000	12.907000	-13.620000
H	-1.366000	8.596000	-11.885000
H	-4.515000	9.279000	-17.383000
H	4.663000	12.672000	-15.846000
H	4.696000	13.694000	-17.304000
H	3.743000	12.186000	-17.288000
H	3.654000	12.267000	-11.737000
H	3.522000	10.821000	-10.716000
H	2.634000	12.285000	-10.289000
H	-2.320000	6.345000	-12.799000
H	-4.086000	6.261000	-13.015000
H	-3.378000	7.315000	-11.774000
H	-5.102000	10.365000	-19.404000
H	-3.903000	9.968000	-20.656000
H	-4.578000	11.605000	-20.570000
H	2.401000	14.235000	-18.292000
H	3.183000	14.680000	-15.572000
H	1.961547	16.476359	-17.288313
